# Identification of high-confidence human poly(A) RNA isoform scaffolds using nanopore sequencing

**DOI:** 10.1261/rna.078703.121

**Published:** 2022-02

**Authors:** Logan Mulroney, Madalee G. Wulf, Ira Schildkraut, George Tzertzinis, John Buswell, Miten Jain, Hugh Olsen, Mark Diekhans, Ivan R. Corrêa, Mark Akeson, Laurence Ettwiller

**Affiliations:** 1 Biomolecular Engineering Department, UC Santa Cruz, California 95064, USA; 2 New England Biolabs, Ipswich, Massachusetts 01938, USA; 3 Genomics Institute, UC Santa Cruz, California 95064, USA

**Keywords:** RNA, transcriptomics, nanopore, direct RNA, single-molecule, full-length, transcription start site, RNA isoforms

## Abstract

Nanopore sequencing devices read individual RNA strands directly. This facilitates identification of exon linkages and nucleotide modifications; however, using conventional direct RNA nanopore sequencing, the 5′ and 3′ ends of poly(A) RNA cannot be identified unambiguously. This is due in part to RNA degradation in vivo and in vitro that can obscure transcription start and end sites. In this study, we aimed to identify individual full-length human RNA isoforms among ∼4 million nanopore poly(A)-selected RNA reads. First, to identify RNA strands bearing 5′ m^7^G caps, we exchanged the biological cap for a modified cap attached to a 45-nt oligomer. This oligomer adaptation method improved 5′ end sequencing and ensured correct identification of the 5′ m^7^G capped ends. Second, among these 5′-capped nanopore reads, we screened for features consistent with a 3′ polyadenylation site. Combining these two steps, we identified 294,107 individual high-confidence full-length RNA scaffolds from human GM12878 cells, most of which (257,721) aligned to protein-coding genes. Of these, 4876 scaffolds indicated unannotated isoforms that were often internal to longer, previously identified RNA isoforms. Orthogonal data for m^7^G caps and open chromatin, such as CAGE and DNase-HS seq, confirmed the validity of these high-confidence RNA scaffolds.

## INTRODUCTION

Most human genes encode multiple transcript isoforms. These isoforms are derived from alternative splicing, alternative transcription start sites (TSS), or alternative transcription termination sites (TTS). TSSs outnumber genes (Forrest et al. 2014), and together with alternate TTS, account for most transcript isoform differences between tissues (Reyes and Huber 2018). Accurate identification of an RNA isoform is difficult when either its TSS or its TTS is unknown or positioned within the genomic region of a larger isoform (Conesa et al. 2016), and internal isoforms are often omitted from transcriptome annotations (Frankish et al. 2019). Direct sequencing of nucleotides between the 5′ cap and 3′ poly(A) tail on individual RNA molecules would reveal the isoform structure and associated modifications without the need for inference-based computational models (Roberts et al. 2011; Mezlini et al. 2013; Kovaka et al. 2019; Tang et al. 2020).

Nanopore RNA sequencing is a single-molecule technique that reads RNA directly rather than cDNA copies. This avoids cDNA artifacts such as PCR amplification bias (Aird et al. 2011), reverse transcriptase (RT) template switching (Cocquet et al. 2006), incomplete reverse transcription due to enzyme failure (Zhang et al. 2001), or due to RNA modifications (Cozen et al. 2015). Furthermore, RNA modifications, which are mostly lost by cDNA synthesis, can be directly detected using nanopore sequencing. Thus far, *N*^6^-methyladenosine (m^6^A) (Garalde et al. 2018; Workman et al. 2019; Parker et al. 2020), inosine (Workman et al. 2019), pseudouridine (Smith et al. 2019), and *N*^7^-methylguanosine (m^7^G) (Smith et al. 2019) have been documented by direct RNA nanopore sequencing. The standard direct RNA nanopore sequencing protocol uses an adapter that is ligated to the poly(A) tail and proceeds to continuously read RNA strands in the 3′-to-5′ orientation. However, reads using the standard direct RNA nanopore protocol terminate before reaching the 5′ end of captured molecules (Workman et al. 2019). RNA strand breaks that occur in vivo, in vitro, or due to signal processing errors separate the adapted 3′ end from the bona fide 5′ end, introducing a truncated 5′ end to the read. Additionally, the standard direct RNA nanopore sequencing protocol has no marker to identify the native 5′ end, because ∼11 nt are lost from the 5′ end of each read when the motor enzyme releases the strand (Workman et al. 2019), further complicating the identification of TSS.

Akin to other techniques targeting mRNA TSS (Adiconis et al. 2018), the m^7^G cap (Furuichi 2015) is a unique feature of mRNA 5′ ends that could be used as a marker for full-length reads. The Oligo-capping method (Kazuo and Sumio 1994), for instance, removes the 5′ cap using an enzyme and then ligates the mRNA to an oligonucleotide adapter followed by reverse transcription into cDNA. Two prior studies demonstrated that combining the Oligo-capping method (Kazuo and Sumio 1994) with direct RNA nanopore sequencing improved TSS identification (Jiang et al. 2019; Parker et al. 2020). However, it has been previously demonstrated that Oligo-capping has the lowest sensitivity and precision of six established methods for determining the TSS (Adiconis et al. 2018). Previously, we used ReCappable-seq to replace m^7^G caps with biotin-modified caps to enrich for native 5′ mRNA ends, which enabled the identification of high-confidence TSS using Illumina sequencing (Yan et al. 2019).

In this study, we introduce a new chemo-enzymatic method, Nanopore ReCappable sequencing (NRCeq), that uniquely and specifically replaces m^7^G caps with azido-modified caps to facilitate their chemical adaptation with an RNA oligonucleotide. This approach allows for sequencing individual RNA molecules containing the poly(A) tail through the 5′ cap, which we define as full-length RNA isoform scaffolds. NRCeq avoids common shortfalls of ligation methods, such as sequence bias (Fuchs et al. 2015; Dard-Dascot et al. 2018) and ligation at 5′ truncated ends. We used NRCeq to experimentally identify individual full-length high-confidence RNA scaffolds in the B-lymphocyte cell line GM12878 poly(A) RNA transcriptome. These scaffolds extended from the m^7^G cap to a documented poly(A) site, including 4876 scaffolds of previously unannotated isoforms. We systematically correlated these scaffolds with orthogonal data, including 5′-RACE, CAGE, DNase-seq, and other markers of TSS validation, and showed that single reads could provide compelling evidence for bona fide RNA isoforms.

## RESULTS

### NRCeq strategy

**FIGURE 1.**
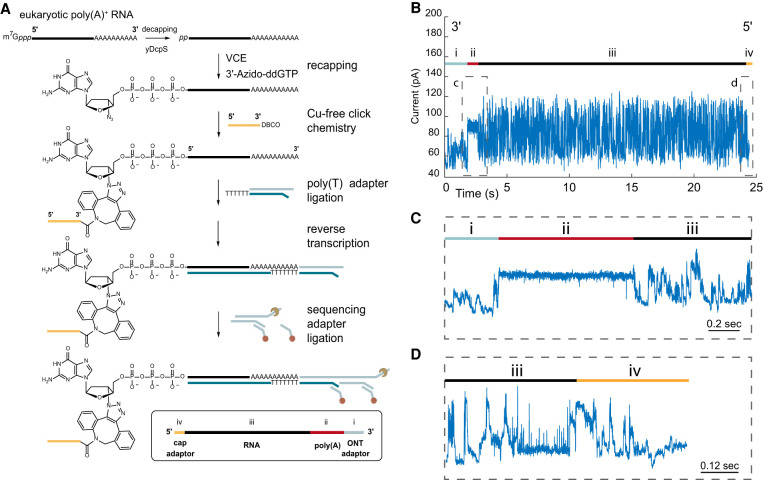
Preparation and analysis of 5′ cap-adapted poly(A) strands. (*A*) Adaptation and library preparation workflow for poly(A)-selected RNA. (*B*) Representative ionic current trace for a cap-adapted full-length RNA read is shown for the thymidine phosphorylase gene (*TYMP*). The trace begins with ionic current associated with the ONT adapter (i). This is followed by a monotonic ionic current associated with the 3′ poly(A) tail (ii) and then a variable ionic current associated with the RNA transcript nucleotides (iii). The final segment is an ionic current signature characteristic of the 45 nt RNA cap-adapter (iv). (*C*) An approximately two second window centered on the ionic current associated with the poly(A) tail (ii). (*D*) An approximately one second window centered on the boundary between the ionic current associated with the 5′ end of the transcript (iii) and a characteristic adapter ionic current trace (iv).

The NRCeq strategy is diagrammed in Figure 1A. First, we used the yeast scavenger decapping enzyme (yDcpS) (Liu et al. 2002; Wulf et al. 2019) to remove the m^7^G cap from poly(A)-enriched RNA, leaving 5′-diphosphate ends. Second, the 5′-diphosphate RNA strands were recapped with 3′-azido-ddGTP using *Vaccinia* capping enzyme (Supplemental Fig. 2; Wulf et al. 2019). Third, the 3′-azido recapped RNA strands were covalently attached to a dibenzocyclooctyne (DBCO) group on the 3′ end of a 45 nt long RNA oligonucleotide adapter using a specific copper-free “click” chemistry reaction (Supplemental Fig. 3; Muttach and Rentmeister 2016; Zhang et al. 2019). The remaining steps in the library preparation follow a typical Oxford Nanopore Technologies (ONT) workflow for poly(A)-selected RNA 3′ adaptation.

A typical cap-adapted ionic current trace for the human thymidine phosphorylase (*TYMP*) gene is shown in Figure 1B. Following strand capture, a characteristic ionic current is caused by translocation of the ONT 3′ adapter (i). This is followed by a monotonic ionic current associated with the 3′ poly(A) tail (ii) and then a variable ionic current with a bottle brush appearance associated with a mixed series of RNA nucleotides (iii). The trace terminated with an ionic current signature characteristic of the 45 nt RNA cap-adapter (iv). This signature indicated that individual strands were read from the 3′ poly(A) tail (Fig. 1C) through the original 5′-capped end (Fig. 1D). We used a sequence-based barcode identification software, Porechop, to detect the adapter on individual nanopore reads (see Materials and Methods).

### Optimizing 5′ cap-adaptation using *Saccharomyces cerevisiae* poly(A) RNA

We optimized the 5′ cap-adaptation strategy using an *S. cerevisiae* poly(A) RNA. The yeast transcriptome is well-suited for this because the m^7^G cap is identical to the human m^7^G cap, and the transcriptome is small and relatively simple compared to other eukaryotes (Botstein and Fink 2011). Initially, we used a copper-catalyzed click reaction for the 5′ adaptation step (Supplemental Methods); however, RNA degradation was unacceptable as measured by RNA integrity number (RIN) (Supplemental Table 2; Schroeder et al. 2006). As an alternative, we implemented a copper-free chemistry step based on a strain-promoted click reaction (Fig. 1A, Materials and Methods; Shelbourne et al. 2011; Zhang et al. 2019). This eliminated RNA degradation during the click step (Supplemental Table 2). Further, this change from copper-catalyzed to copper-free chemistry improved the percentage of yeast poly(A) RNA reads that were cap-adapted (13.4%–38.4%, respectively) and the read N50 (692–744 nt, respectively), the latter being a measure where half of the total bases sequenced are contained in reads of that length or longer.

### Applying NRCeq to human poly(A) RNA transcripts

Having optimized 5′ cap-adaption chemistry and detection, we applied this strategy to poly(A) RNA isolated from GM12878 cells, a model human B-lymphocyte cell line. We acquired four million reads with quality scores greater than or equal to 7 that went through the cap-adaptation process (we refer to this population as “treated reads” in the text that follows, see Supplemental Fig. 7a). We identified 574,091 (14.3%) of the treated reads as “cap-adapted” (see Materials and Methods, Supplemental Figs. 1, 7b). As a control, we also performed standard native RNA nanopore sequencing using the same starting poly(A) RNA material (“untreated reads”) yielding approximately 3.8 million reads from two replicates (Supplemental Table 1).

**FIGURE 2.**
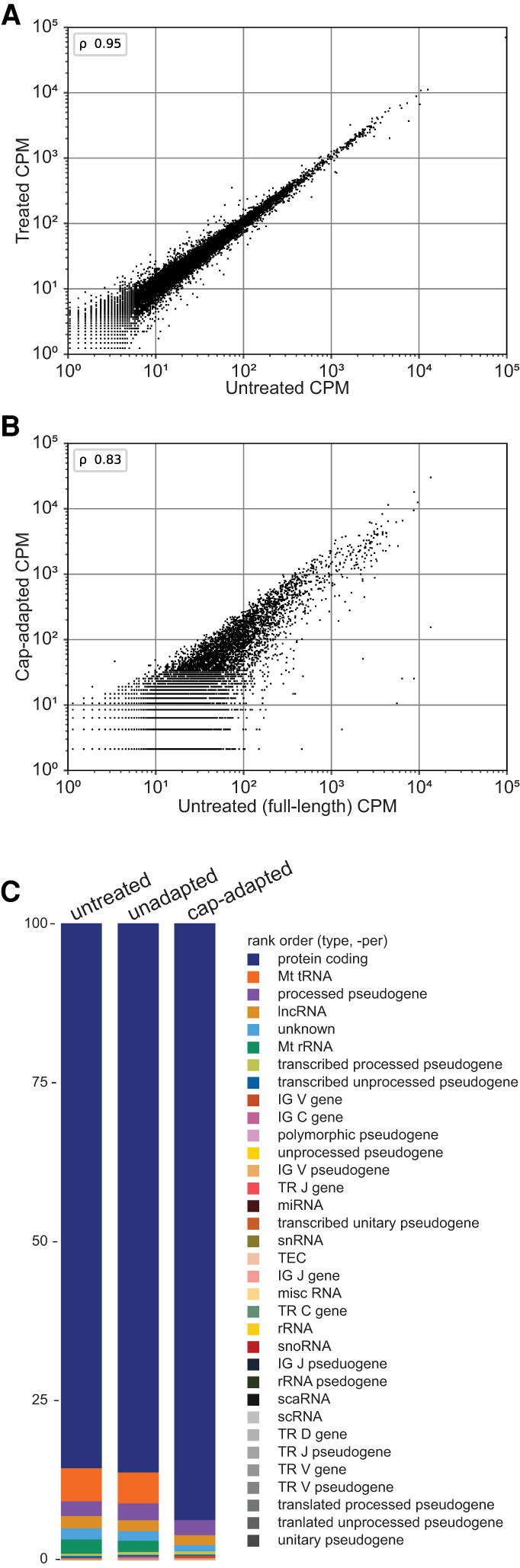
Cap-adaptation performance on GM12878 RNA. Number of transcripts per gene counts per million (CPM) correlation plots (*A*) between untreated and treated samples, and (*B*) between full-length untreated and cap-adapted samples. Pearson's *r* (ρ) were 0.95 and 0.83, respectively. (*C*) Percent of RNA by class for untreated, unadapted, and cap-adapted reads. Unadapted refers to reads within the treated samples that are missing the adapter sequence. All class percentages are in Supplemental Table 3.

The N50 value for the cap-adapted reads was 1301 nt, which was shorter than the N50 value for untreated reads (1614 nt) (Supplemental Table 1). Given this difference, we were concerned that the cap-adaptation process biased the overall results either by inducing more RNA strand breaks or by removing longer RNAs from the sample. To test this, we compared the number of transcript copies per gene for the untreated and treated samples. The Spearman rank correlation score was very strong (0.95), indicating that the treatment protocol did not substantially alter the RNA composition of the sample (Fig. 2A). We then compared the number of transcript copies per gene for the cap-adapted and full-length untreated samples using a previously described definition for full-length (the 5′ end of the read is within 25 bases of the annotated 5′ end and the 3′ end of the read is within 50 bases of the annotated 3′ end) (Smith et al. 2019; Workman et al. 2019). The Spearman rank correlation score was lower, but also very strong (0.83) (Fig. 2B). This suggested that the cap-adaptation process induced RNA strand breaks, which is also supported by the RIN analysis performed with yeast (Supplemental Table 2), but that the overall impact on transcript detection was minor.

Among untreated and cap-adapted reads, 85% and 94% of aligned nanopore reads, respectively, corresponded to protein-coding genes (Fig. 2C). Mitochondrial RNA reads accounted for 11% of those mapped untreated reads. By comparison, mitochondrial RNA reads account for only 0.3% of the mapped cap-adapted reads. This result was expected because the NRCeq procedure can adapt triphosphates, diphosphates, and m^7^G capped 5′ ends, but mitochondrial transcript 5′ ends usually bear a 5′ monophosphate or are transient, with a subset capped by NAD^+^ and NADH (Bird et al. 2018). When we screened for preprocessed ribosomal RNA, which bears triphosphate 5′ ends, we found that only 11 out of 574,091 total cap-adapted reads aligned to ribosomal RNA genes.

### TSS identification with NRCeq

**FIGURE 3.**
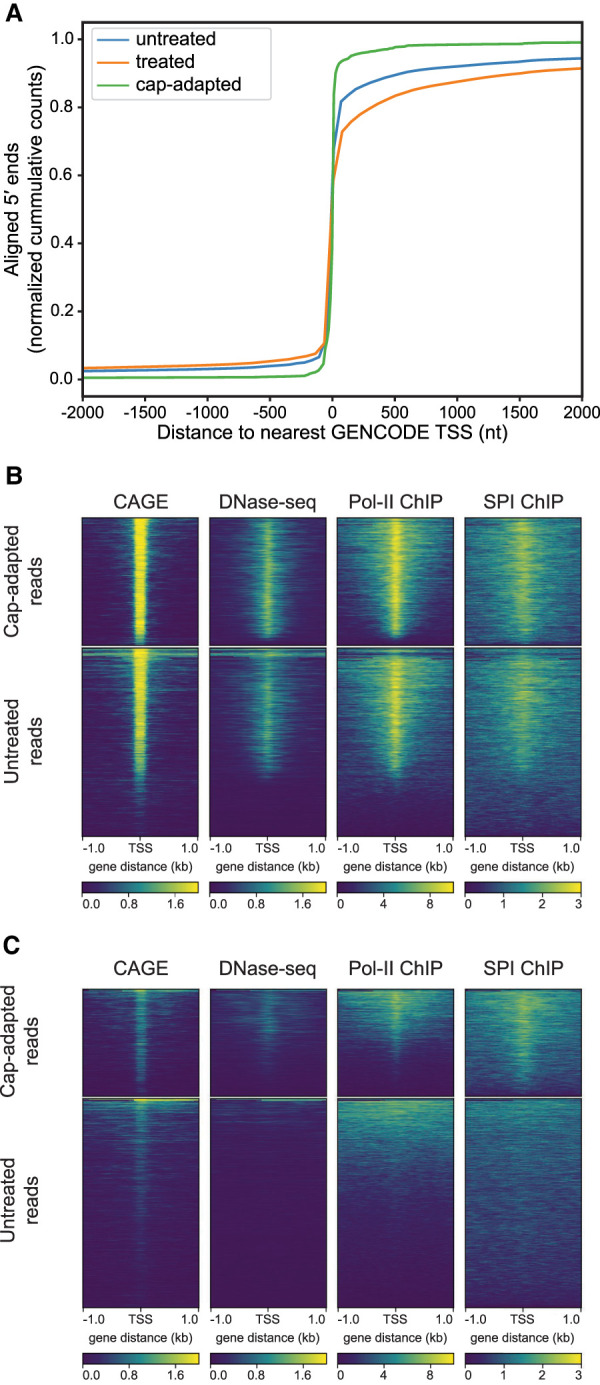
Correspondence between RNA nanopore 5′ end reads and orthogonal TSS evidence. (*A*) Nucleotide distance of RNA 5′ ends from annotated GENCODE TSS. The *x*-axis is the number of nucleotides between a nanopore read 5′ end and the closest TSS annotated in GENCODE v.32. Negative numbers are upstream (5′) from the TSS; positive numbers are downstream (3′) from the TSS. The *y*-axis is the cumulative number of RNA nanopore reads at a given distance of their 5′ end from an annotated GENCODE TSS. Values are normalized as a fraction of total counts for a given treatment. (*B*) Comparison of poly(A) RNA nanopore 5′ end reads to orthogonal TSS markers. Each plot is a heatmap where the *x* axis is a ±1 kb window centered on the genomic position defined by the 5′ end of each RNA nanopore read. Each row in the *y*-axis is a genomic region defined by individual reads. The color intensity is the read depth normalized signal (CAGE, DNase-seq) or fold change over control for each position (POLR2 and SPI1). The *top* plots are cap-adapted reads, the *bottom* plots are untreated reads. (*C*) Comparison of unannotated RNA nanopore 5′ end reads to orthogonal TSS markers. Unannotated 5′ ends are defined as positions where the 5′ end of at least two reads are aligned within 50 nt of each other and more than 300 nt away from any annotated TSS. The number of reads in each plot was down-sampled to 9116 reads and then filtered for only unique positions (total number of unannotated cap-adapted reads).

NRCeq was designed to identify m^7^G-capped RNA 5′ ends and improve base calling near those ends. We predicted that cap-adapted nanopore reads would be enriched for 5′ ends proximal to TSS annotated by GENCODE (Frankish et al. 2019). This prediction was substantiated (Fig. 3A). We found that 99% of 5′ ends for cap-adapted reads were within a window of 300 bp of an annotated TSS, compared to 77% of the untreated reads, and 65% of the treated reads (Fig. 3A).

Precise definition of TSS can be difficult. Therefore, we compared the start of the cap-adapted reads to those of other markers that are conventionally used for TSS determination. These included DNase-seq, Pol II ChIP-seq, SPI1 ChIP-seq, and CAGE, all performed on GM12878 cells. We found that a majority of the cap-adapted reads corresponded with these other markers of transcription initiation (Fig. 3B; Supplemental Fig. 9).

It is noteworthy that a low number of the cap-adapted reads had 5′ ends that did not clearly map to annotated TSS, suggesting alternative TSS. To test this hypothesis, we filtered cap-adapted reads whose 5′ ends mapped >300 nt from any annotated GENCODE TSS (Frankish et al. 2019). We found 9116 reads (1% of total) corresponding to 1914 genes. A majority of these newly found 5′ ends were validated by DNase-seq, ChIP-seq, and CAGE (Fig. 3C), thus increasing the confidence that these are bona fide TSS (Adiconis et al. 2018). The same pipeline identified 240,211 untreated reads (20% of total) that corresponded to unannotated 5′ TSS. However, the vast majority of these 5′ TSS were not validated by DNase-seq and ChIP-seq (Fig. 3C). Interestingly, a weak CAGE signal can be observed despite the lack of genomic TSS marks, which could be attributed to noise in the CAGE data set.

### 5′ RACE validation of unannotated TSS

We used 5′ RACE (Frohman et al. 1988) to test the validity of 93 selected isoforms from 88 genes bearing presumptive new TSS (Supplemental Fig. 6). These TSS were chosen because they started either at an internal exon or at an unannotated exon. Two highly expressed genes with documented TSS, *ACTB*, and *TMSB10* were used as positive controls. To eliminate 5′ RACE products arising from uncapped phosphorylated ends, total RNA was first treated with a calf intestinal alkaline phosphatase (CIP). To identify RACE products originating from the position of capped ends, the RNA was subsequently treated with an RNA 5′ pyrophosphohydrolase (RppH) before library preparation, enabling the enzymatic ligation of RNA oligonucleotide adapters to the originally capped 5′ end of transcripts. Finally, to confirm the occurrence of the cap, we demonstrated the dependence of 5′ RACE products on decapping with RppH.

**FIGURE 4.**
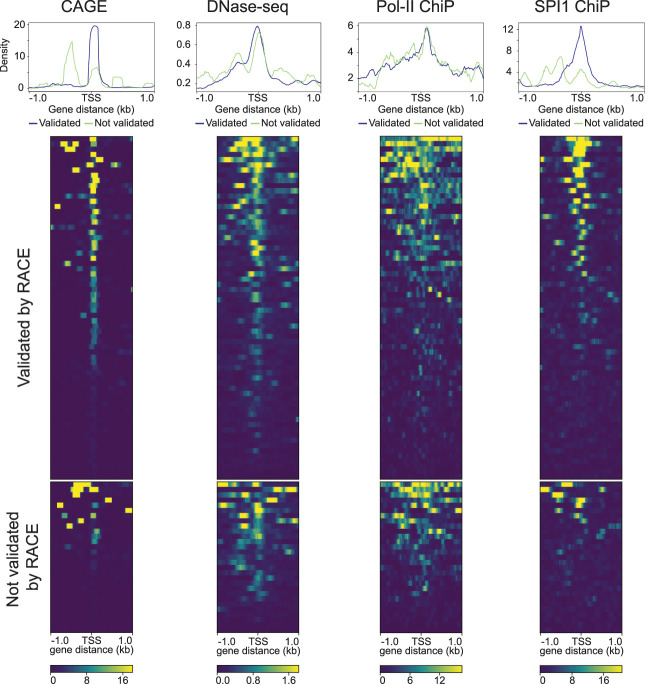
Multiplex RACE. Heatmaps of three promoter marks: PolR2 (ENCFF340BYJ), SPI1 (ENCFF289XSX) ChIP-seq, DNase-seq (ENCFF093VXI), and CAGE (ENCFF580WIH), each showing 2 kb flanking regions at the 5′ end of the 93 candidate isoforms. The *upper* panel shows the 64 candidate TSS that were validated by RACE, the *lower* panel corresponds to the 29 TSS not validated by RACE.

Amplification used a reverse PCR primer 100–200 nt downstream from the unannotated TSS. Short-read 5′ RACE sequencing data confirmed that the TSS for 64 of the 93 target isoforms had a capped RNA 5′ end within 50 nt of the TSS identified by NRCeq (Supplemental Table 4). In addition to the RACE results, CAGE or TSS chromatin marks corresponded with the 5′ end of 64 of the 93 presumptive TSS (Fig. 4). A likely reason that the 29 unconfirmed TSS were not validated by RACE was the lack of optimization for the 93 individual PCR reactions (primer sequence, temperature, buffer).

### Documentation of full-length RNA scaffolds

A central aim of this study was to facilitate mRNA isoform identification using individual full-length reads as scaffolds. This required identification of nanopore reads that aligned to protein-coding genes, and that correctly identified both the 5′ and 3′ ends of mature mRNA. It was possible that some of the 5′ ends in our cap-adapted reads were misaligned by minimap2 (Li 2018). To address this possibility, we filtered the cap-adapted alignments for reads with 15 or fewer 5′ soft-clipped bases. This resulted in 294,107 (7.2% of treated reads, 51.2% of cap-adapted reads) reads that we termed high-confidence alignments (Supplemental Fig. 7c). Additionally, it was possible that the 3′ end of a given read was not the mature 3′ end of the RNA. For example, we observed a small fraction of reads with 3′ ends in the middle of an internal exon. This could be the result of improper signal processing (Workman et al. 2019), RNAs that had poly(A) tails added during biological strand degradation (Slomovic et al. 2010), or an improper ligation event.

**FIGURE 5.**
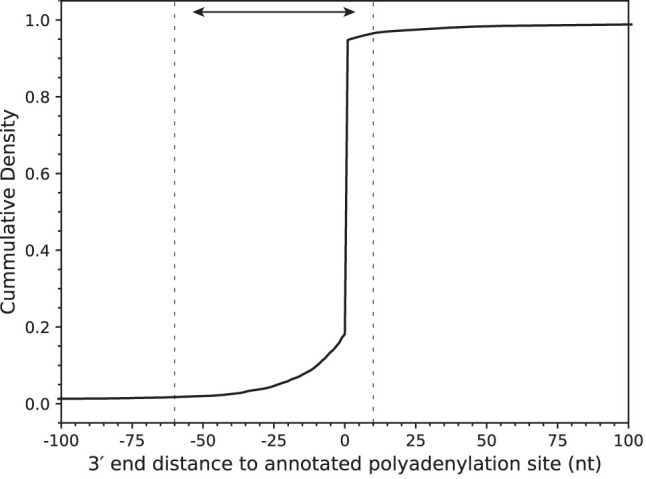
mRNA scaffold 3′ end distance to known polyadenylation site. Reads with aligned 3′ ends upstream of a polyadenylation site (internal to the gene) are represented with negative distances, and downstream from a polyadenylation site (external to the gene) are represented with positive numbers. The dashed lines are at −60 and +10 from an annotated polyadenylation site, 99% of mRNA scaffolds fall within this range.

To address the possibility that there was an improper ligation event or improper signal processing, we screened the high-confidence 294,107 (7.2% of the treated reads, 51.2% of cap-adapted reads) nanopore 5′ capped RNA reads for the presence of poly(A) tails using nanopolish-polya (Workman et al. 2019), which resulted in 222,687 (5.5% of the treated reads, 75.7% of alignment filtered reads) reads. To filter for full-length RNA, we identified capped reads that had 3′ ends that aligned within −60 to +10 nt of annotated polyadenylation sites as recommended by PolyASite 2.0 (Supplemental Fig. 7e; Herrmann et al. 2019). This nucleotide window ensured that we removed partially degraded RNAs that were poly(A) tailed as part of the degradation cycle (Slomovic et al. 2010). This resulted in 209,093 [5.1% of treated reads, 93.9% of poly(A) tail filtered reads] individual full-length poly(A) RNA scaffolds (Fig. 5). There were 195,602 (4.8% of treated reads, 93.5% of polyA site filtered reads) scaffolds that corresponded to 8740 protein-coding genes (Supplemental Fig. 7f). The number of transcripts per protein-coding gene ranged from 1-to-7987 (Supplemental Fig. 5c). Among these, we identified 4876 (0.12% of treated reads, 2.5% of high-confidence scaffolds) full-length RNA scaffolds with unannotated TSS that mapped to protein-coding genes (Supplemental Fig. 7g).

We then performed a statistical measure of confidence for each full-length RNA scaffold using mapping quality scores (Li et al. 2008). These mapping quality scores for minimap2 range from zero (Q0, equal probability that the scaffold aligned to more than one position in the reference genome) to 60 (Q60, ∼1 × 10^−6^ probability that the alignment was in the wrong position). Among the 209,093 RNA scaffolds, 151,182 (72.3%) had mapping quality scores of 60 (Supplemental Fig. 5a). There were 14,282 (6.8%) full-length RNA scaffolds with mapping quality scores of zero. Among the 4876 full-length RNA scaffolds for unannotated isoforms, 4568 (93.7%) had mapping quality scores of 60 (Supplemental Fig. 5b). There were 49 (1.0%) full-length RNA scaffolds with mapping quality scores of zero (Supplemental Fig. 5b). By comparison, the untreated reads had 71.2% of the reads with a mapping quality score of 60.

### NRCeq 5′ ends agree with orthogonal markers of TSSs

Computational tools are often used to predict RNA isoforms using short-read cDNA data. Recently they have also been applied to nanopore direct RNA data (Kovaka et al. 2019; Tang et al. 2020). We reasoned that NRCeq could provide physical evidence for those predictions.

As a test, we compared three orthogonal TSS markers (CAGE, Pol II ChIP-seq, and DNase-HS sites) with 5′ ends predicted by StringTie2, FLAIR, untreated reads, and NRCeq. For each data set, we merged all 5′ ends within a 25 nt window into individual unique 5′ ends. We then compared these unique 5′ ends with the orthogonal TSS markers (Supplemental Table 6). Four results stood out: (i) overlap with the TSS markers was similar for StringTie2 (61.5%), FLAIR (74.7%), and cap-adapted reads (61.5%); (ii) overlap with the TSS markers was also similar for untreated (38%) and NRCeq treated (36%) reads; (iii) overlap with the TSS markers was strong for high-confidence scaffolds (92.2%); and (iv) overlap with Pol II alone was the highest among the three TSS markers for each data set. A possible explanation is that Pol II ChIP-seq covers more of the GM12878 genome (Supplemental Table 7).

Next, we compared the predicted TSS from StringTie2 and FLAIR with TSS for the NRCeq high-confidence scaffolds. The overlap was 26.8% (3591 TSS) for StringTie2 and was 53.9% (7210 TSS) for FLAIR. We found that 21.6% (2894 TSS) unique 5′ ends were shared between all three data sets (Supplemental Fig. 11).

### Use of high-confidence scaffolds to define candidate human mRNA isoforms

We proposed that high-confidence RNA scaffolds could help identify previously unannotated isoforms at sufficient precision to warrant further detailed biological experimentation, as well as detect RNA modifications on individual molecules (Supplemental Fig. 8). The following two examples illustrate a pipeline we used to characterize two unannotated candidate mRNA isoforms.

*Diacylglycerol O-acyltransferase 1 (DGAT1)* encodes a multipass transmembrane protein that catalyzes the conversion of diacylglycerol and fatty acyl CoA to triacylglycerol. There are six annotated isoforms in GENCODE (Frankish et al. 2019) and two annotated isoforms in RefSeq (Supplemental Fig. 4; O'Leary et al. 2016). Among 30 untreated nanopore reads that aligned to *DGAT1*, two reads had 5′ exons that are not documented by GENCODE (Frankish et al. 2019) nor by RefSeq (O'Leary et al. 2016). In neither case was it possible to determine if the 5′ ends represented a mature mRNA transcript or a truncation product. Importantly, a single high-confidence mRNA scaffold corresponded to one of these presumptive isoforms. This internal TSS also had a signal in CAGE and DNase-HS data from GM12878, which without the full-length transcript information could easily be mistaken as noise. This confirmed connectivity between an m^7^G cap, the unannotated first exon, 17 exons present in known isoforms, and a confirmed poly(A) tail.

**FIGURE 6.**
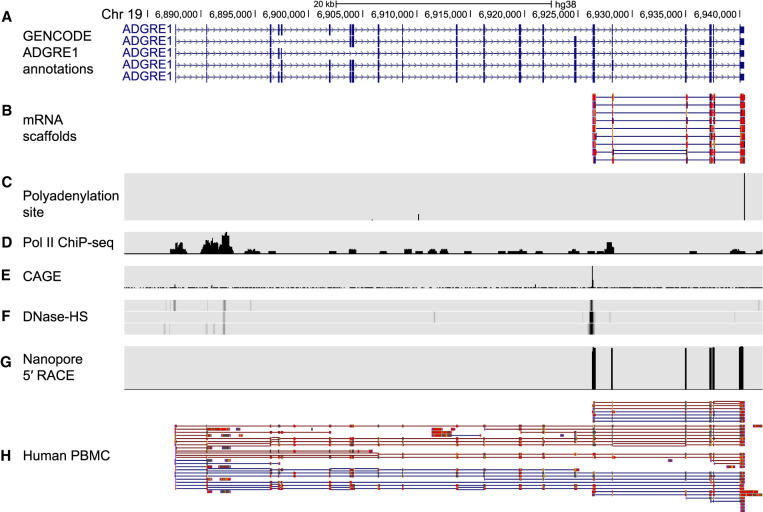
mRNA scaffolds predict an unannotated *ADGRE1* isoform. (*A*) GENCODE v32 annotations for *ADGRE1* mRNA isoforms. (*B*) Nine high-confidence mRNA scaffolds for an unannotated *ADGRE1* isoform. (*C*) Polyadenylation sites annotated by the Poly(A)Site 2.0 atlas (Herrmann et al. 2019). GM12878 specific data from: (*D*) Pol II ChIP-seq sites (Barski et al. 2007); (*E*) CAGE sites for the positive strand (Djebali et al. 2012); (*F*) three replicate DNase-HS tracks (Davis et al. 2018). (*G*) Read coverage from full-length nanopore 5′ RACE sequencing. (*H*) Human peripheral blood mononuclear cell (PBMC) nanopore cDNA reads (Volden et al. 2018). Red and blue lines indicated forward and reverse alignments, respectively.

*Adhesion G protein-coupled receptor E1 (ADGRE1)*. ADGRE1 is a class II adhesion GPCR that is expressed in differentiated cells in the human myeloid lineage (Waddell et al. 2018). ADGRE1 is often used as a biomarker for macrophages; however, its function is unknown (Waddell et al. 2018). The five annotated human isoforms (Fig. 6A) encode proteins with extracellular EGF-like binding domains and 7-transmembrane domains (McKnight and Gordon 1998).

In our high-confidence cap-adapted data set, each of nine individual RNA scaffolds aligned to a proposed ∼1100 nt long unannotated isoform of *ADGRE1* (Fig. 6B). This proposed isoform had a TSS that was internal to the annotated *ADGRE1* isoforms. The nine scaffolds included six previously documented exons, that together encoded an in-frame ORF consistent with a protein composed of transmembrane domains 3-to-7 of the annotated ADGRE1 receptors. The extracellular amino terminus and transmembrane domains 1 and 2 were absent in the isoform predicted by the nine scaffolds.

The expected median identity is 87% for nanopore RNA sequencing reads (Workman et al. 2019). Consequently, additional information would be needed to establish a high-confidence isoform based on a single nanopore mRNA scaffold. We confirmed that the mapping quality score for each of the scaffolds was 60, and there was a canonical poly(A) site proximal to the 3′ ends (Fig. 6C; Herrmann et al. 2019). We found that Pol II ChIP-seq, CAGE, and DNase-seq data all supported the proposed unannotated ADGRE1 isoform (Fig. 6D–F). Since these other features were consistent with the presence of an unannotated TSS, we performed an *ADGRE1*-targeted template-switching 5′ RACE experiment using nanopore sequencing (see Materials and Methods). The resulting cDNA reads confirmed the 5′ end and revealed amplicons with identical exon composition as the RNA nanopore scaffolds (Supplemental Fig. 7g).

It was possible that the isoform was an artifact specific to the immortalized GM12878 cell line. To rule this out, we examined long-read cDNA sequencing data from primary human peripheral blood mononuclear cells (PBMC) (Volden et al. 2018). Seven out of nearly 50 reads that aligned to *ADGRE1* were identical to the unannotated isoform identified by the nanopore scaffolds (Fig. 6H). This indicated that the proposed isoform was not specific to GM12878 cells.

## DISCUSSION

In this study, we describe a strategy that uses individual nanopore reads to define high-confidence human poly(A) RNA scaffolds. These scaffolds include the 5′ m^7^G cap, the 3′ polyadenylation site, and the intervening sequence at 87% identity. A majority of these scaffolds had a mapping quality score of Q60. Most of these scaffolds (95%) confirmed isoforms previously annotated in GENCODE v32 (Frankish et al. 2019). There were also 4876 full-length RNA scaffolds whose TSS were not annotated in GENCODE v32 (Frankish et al. 2019), a majority of which were validated by orthogonal transcription initiation markers. Most of these TSS were internal to known mRNA isoforms.

This strategy includes a new chemo-enzymatic method to specifically adapt 5′ capped RNA strands. The RNA oligonucleotide component of the cap-adapter permitted both identification of the biological 5′ end and sequencing of approximately six additional nucleotides that were systematically missed using the conventional ONT RNA sequencing protocol. Due to the nature of the cap-adapter linkage used by NRCeq, approximately five nucleotides are still missing at the 5′ end of each strand, including the N1 and N2 positions which are often modified (Furuichi 2015). For this reason and the 87% RNA base call percent identity of direct RNA nanopore sequencing, isoforms identified by NRCeq are better defined as RNA scaffolds. Improvements to the technology will be made possible both through redesigning the cap-adapter linkage to prevent unnecessary motor enzyme slippage, as well as increased accuracy of RNA base calls from ONT. We anticipate improvements to RNA nanopore sequencing accuracy because DNA base call accuracy has increased from ∼66% in 2014 (Jain et al. 2015) to ∼96.4% in 2020 (unpublished UCSC data).

Although only 14.3% of reads were identified as cap-adapted, the actual adaptation efficiency is likely much higher. The chemo-enzymatic process was demonstrated to be quantitative with synthetic RNAs (Supplemental Figs. 2, 3), suggesting that other factors may contribute to the lower percentage of cap-adapted reads. NRCeq does not eliminate RNA strand breaks that separate the adapted 3′ end from the native 5′ end. Therefore, the cap-adapter on several of the RNA 5′ ends does not get detected because of these truncations. This results in a lower observed cap-adaptation percentage than might otherwise be expected based on the efficiency of the chemo-enzymatic process. Minor changes to RNA handling or samples with higher population of shorter average transcript lengths are likely to show an improved cap-adaptation efficiency. Additionally, despite these truncations, non-cap-adapted reads are not lost using NRceq, which can still be used to inform predictions about RNA isoforms and expression.

The 5′ adaptation procedure described in this study can also be used to attach an oligonucleotide to particular 5′ termini not originating from an m^7^G cap. Alternative 5′ termini, such as triphosphate can be adapted through NRCeq in addition to an m^7^G cap (Yan et al. 2019). Therefore, it is conceivable that the poly(A) RNA data set contains transcripts produced by RNA polymerases other than Pol II. However, given our filtering criteria and poly(A) enrichment step, it is unlikely that non-Pol II transcripts were identified by NRCeq in this study. But it is an important feature of NRCeq to consider when using other experimental conditions. Importantly, 5′ termini bearing a monophosphate are not adapted using NRCeq. This eliminates the possibility of adapting 5′ ends resulting from RNA processing or strand breaks, which can obscure TSS observations.

While other methods facilitate TSS identification using direct RNA nanopore sequencing, NRCeq provides several improvements. The cap-adaptation strategy used in NRCeq is more reliable than ligation-based methods that can be biased (Fuchs et al. 2015; Dard-Dascot et al. 2018). Methods such as Oligo-capping (Kazuo and Sumio 1994) may be subject to false TSS identification due to 5′ monophosphate RNA contamination if CIP treatment is incomplete prior to ligation. These 5′ monophosphate RNAs are not adapted by NRCeq. Importantly, adaptation rates for NRCeq, and previously reported Nanopore Oligo-capping strategies are similar (14%–40% for NRCeq, ∼26% for Parker et al. 2020), 17.8% for Jiang et al. (2019), despite using different RNA sources (human, yeast, arabidopsis, and locust). Aside from experimental methods, computational predictions of isoforms are possible with programs such as StringTie2 (Kovaka et al. 2019) and FLAIR (Tang et al. 2020). The disadvantage is that these predictions often lack concrete physical evidence for the TSS. NRCeq can provide that evidence because it labels m^7^G capped RNA molecules.

In this study, we used GENCODE v32 (Frankish et al. 2019) to identify annotated and unannotated isoforms in our high-confidence poly(A) RNA scaffold data. This gene model set is the method of choice for high-throughput RNA analysis. RefSeq is an alternative gene model set that is often used for human genetics (O'Leary et al. 2016). The unannotated *ADGRE1* exemplar was absent in both gene models. However, in other cases, we found annotations in RefSeq (O'Leary et al. 2016) that matched isoforms from our nanopore data that were absent in GENCODE v32 (Frankish et al. 2019). Two examples are Profilin 1 (*PFN1*) and Voltage Dependent Anion Channel 1 (*VDAC1*). We recommend comparing proposed unannotated isoforms against both of these gene model sets.

We identified an unannotated *ADGRE1* isoform with an internal TSS and an in-frame ORF with the annotated isoforms. We substantiated this proposed *ADGRE1* isoform using four criteria that could be broadly applied to any target scaffold: (i) establish that the alignments appear real (alignment quality scores) using alignment visualization software; (ii) confirm the TSS and TTS correspond with orthogonal markers of these genomic features; (iii) identify the isoform in a follow-up orthogonal experiment; and (iv) determine whether the isoform is a cell line artifact by comparing to long-read RNA-seq of primary tissue. These criteria can be completed in a few hours to a few days, increasing the viability of rapidly annotating new isoforms. However, unambiguous proof that this and other proposed mRNA isoforms are translated by the ribosome will require protein evidence.

In summary, NRCeq enabled identification of individual, high-confidence, RNA scaffolds representing annotated, and unannotated full-length human RNA isoforms.

## MATERIALS AND METHODS

### Synthesis of 3′-DBCO RNA adapter

The 45-nt 3′-DBCO RNA oligomer (CUCUUCCGAUCUACACUCUUUCCCUACACGACGCUCUUCCGAUCU) was synthesized by coupling the 3′-NH_2_ RNA oligomer with a DBCO-sulfo-NHS ester (Glen Research, #50-1941). The 3′-NH_2_ RNA synthesis was performed on an ABI 394 DNA synthesizer (Applied Biosystems) starting with 3′-PT-amino-modifier C3 CPG (Glen Research, #20-2954) and UltraFast RNA TBDMS RNA amidites (Glen Research: Bz-A-CE #10-3003, Ac-C #10-3015, Ac-G-CE #10-3025, and U-CE #10-3030). The oligonucleotide was deprotected according to the manufacturer's protocol using ammonium hydroxide/methylamine and purified using a Glen-Pak RNA purification cartridge (Glen Research, #60-6100) followed by PAGE. The purified 3′-NH_2_ RNA was dissolved in 5 mL of 0.1 M sodium borate (pH 8.3). Then, 2.5 mL of a 20 mM solution of DBCO-sulfo-NHS ester in DMSO was added and stirred for 1.5 h at room temperature. The reaction was then dissolved in 0.1 M TEAB (up to 35 mL) and purified by C8 RP-HPLC (Higgins Analytical) using 0.1 M TEAB and acetonitrile as mobile phase. The 3′-DBCO RNA oligonucleotide was concentrated and repurified by PAGE and desalted using a Clarity-RP desalting cartridge (Phenomenex, #8B-S041-HBJ).

### 5′ RACE library

Total RNA from GM12878 (12 µg) was treated with QuickCIP (NEB, #M0525) at 0.5 U/µL in the provided buffer at 37°C for 20 min and then purified using RNA Clean & Concentrator (Zymo Research, #R1013) with the standard protocol. The RNA was divided into two aliquots (1 and 2). Aliquot 1 was treated with RppH (NEB, #M0356) in 1× Thermopol Buffer (NEB, #B90004) at 0.5 U/µL at 37°C for 1 h, whereas aliquot 2 was incubated under the same conditions in the absence of RppH to serve as a non-decapped control. The two samples were purified as above and eluted in 20 µL of RNase-free water. Each sample (10 µL) was ligated to 5 pmol of a single-stranded RNA adapter (see below) using 1.5 µL T4 RNA Ligase I (NEB, #M0204) in 1× T4 RNA Ligase Reaction Buffer in 20 µL total volume for 1 h at 25°C. Sample 1 (RppH treated) was ligated to the adapter SRGAUUA and sample 2 (mock treated) to adapter SRAUCAG, wherein SR denotes the sequence: GUUCAGAGUUCUACACUCCGACGAUC. The 3′ terminal five nucleotides of each adapter served as an identification index for the provenance of the sequence products (sample 1, decapped vs. sample 2, not decapped). After ligation the two samples were pooled and mixed with 80 µL AMPure XP magnetic beads (Beckman Coulter) and processed for magnetic purification of the RNA, which was eluted in 20 µL water. The RNA was used for first strand cDNA synthesis with random priming using the Protoscript II First Strand Synthesis Kit (NEB, #E6560) in a total volume of 40 µL for 1 h at 42°C. The cDNA was diluted to 150 µL and aliquoted into 96 wells of a PCR plate (1.5 µL/well), each of which contained 20 pmol of a reverse primer specific for each target sequence (see Supplemental Table 5 for target genes and primer sequences). The PCR reactions were performed using the forward primer AATGATACGGCGACCACCGAGATCTACACGTTCAGAGTTCTACAGTCCGA, and LongAmpTaq (NEB, #M0287) with the following program: 94°C 1 min, followed by five cycles of 94°C 10 sec, 60°C 15 sec, 65°C 15 sec, followed by 32 cycles of 94°C 10 sec, 55°C 15 sec, 65°C 15 sec, followed by 65°C 5 min. An aliquot from each reaction (2 µL) was evaluated for quality and to estimate product concentration using the Agilent 2200 TapeStation system. A fraction of each reaction (varying from 1 to 10 µL depending on concentration) was used for pooling to obtain a mixture of all 96 PCR products. The DNA mixture was purified using 200 µL AMPure XP beads per 125 µL DNA, eluted in 40 µL water. Illumina adapters were added by amplifying the sample for four cycles using the SR primer and index primer from the kit (NEB, #E7330). The resulting product was purified with AMPure XP beads at 1:1 ratio, and the eluted material was sequenced in an Illumina MiSeq sequencer using paired end 2 × 150 nt. The FASTQ files were processed to identify the 5′ end terminal sequence of each targeted transcript. RppH treated and untreated reads were distinguished using Cutadapt (version 2.10) with the following parameters: cutadapt -O 5 –action lowercase –trimmed-only –pair-filter first and -g ^GATTA (for reads from the RppH treated sample) and -g ^ATCAG (for reads from the untreated sample). The resulting paired end reads were trimmed using Cutadapt wrapped with trim galore (version 0.3.3) (Martin 2011). The trimmed paired end reads were aligned to the human genome using STAR_2.4.0b (Dobin et al. 2013) using default parameters, and the mapped read 2 was discarded. The ratio of the number of R1 reads from the RppH treated sample over the sum of reads from both samples mapping within a 100 bp window of a predicted TSS position was calculated. For a TSS to be considered validated, a ratio higher than 0.5 was required with at least one RACE read from the RppH treated sample.

### GM12878 cell tissue culture

GM12878 cells were cultured the same as in Workman et al. (2019). Briefly, GM12878 cells (passage 11) were cultured in RPMI medium (Gibco, #21870076) supplemented with 15% non-heat-inactivated FBS (Gibco, #12483020) and 2 mM L-Glutamax (Gibco, #35050061). Cells were expanded to 9 × T75 flasks (45 mL of medium in each) and centrifuged for 10 min at 100*g* (4°C), washed in 1/10th volume of PBS (pH 7.4), and combined for homogeneity. The cells were then evenly split between 8 × 15 mL tubes and pelleted at 100*g* for 10 min at 4°C. The cell pellets were then snap frozen in liquid nitrogen and immediately stored at −80 °C.

### Isolation of GM12878 total RNA

GM12878 RNA was isolated the same as in Workman et al. (2019). Briefly, 4 mL of TRI-Reagent (Invitrogen, #AM9738) was added to a frozen pellet of 5 × 10^7^ GM12878 cells and vortexed immediately. This sample was incubated at room temperature for 5 min. CHCl_3_ (chloroform, 200 µL) was added per mL of sample, vortexed, incubated at room temperature for 5 min, vortexed again, and centrifuged for 10 min at 12,000*g* (4°C). The aqueous phase was pooled in a LoBind Eppendorf tube and combined with an equal volume of isopropanol. The tube was mixed, incubated at room temperature for 15 min, and centrifuged for 15 min at 12,000*g* (4°C). The supernatant was removed, the RNA pellet was washed with 750 μL 80% ethanol and then centrifuged for 5 min at 12,000*g* (4°C). The supernatant was removed. The pellet was air-dried for 10 min, resuspended in nuclease-free water (100 μL final volume), quantified, and either stored at −80°C or processed further by Poly(A) purification.

### GM12878 poly(A) RNA purification

Poly(A) RNA was purified from GM12878 total RNA with NEXTflex poly(A) beads (Bioo Scientific, NOVA-512980) using 50 µL of beads per 100 µg of total RNA. GM12878 poly(A) RNA was aliquoted and stored at −80°C.

### Isolation of *S. cerevisiae* S288C total RNA

Total RNA was purified from *S. cerevisiae* S288C. The *S. cerevisiae* was grown in 1 L YPD media (1% yeast extract, 2% peptone, 2% dextrose) at 30°C. The cells were pelleted and resuspended in cold 10 mM EDTA. The cells were again pelleted and resuspended in 5 mL of 50 mM sodium acetate (pH 5.5), 10 mM EDTA, 1% SDS. An amount of 5 mL of Acid-Phenol:Chloroform:IAA (Invitrogen, #AM9720) was added, and the mixture was vortexed. The mixture was incubated in a 65°C water bath with brief vortexing every 5 min for a total incubation time of 30 min. The mix was placed on ice for 10 min, and the phases separated by centrifugation. The upper phase was collected, and an equal volume of chloroform was added. The mixture was vortexed again, and the phases separated by centrifugation. The upper phase was collected, and 0.1 volume of 3 M sodium acetate pH 5.3 was added. An equal volume of isopropanol was added, mixed, and the RNA was precipitated at −20°C. The resulting RNA precipitate was dissolved in 5 mL of TE buffer. The RNA was reprecipitated by adding 0.25 volume of 1 M sodium acetate pH 5.5 and 2.5 volumes of ethanol and incubated for 60 min at −20°C. The total RNA pellet was redissolved in TE buffer.

### *S. cerevisiae* S288C poly(A) RNA purification

Poly(A) RNA was isolated from 2 mg of total *S. cerevisiae* RNA using the PolyA Spin mRNA Isolation Kit (NEB, #S1560). After a single round of isolation, the RNA was precipitated by adding glycogen and 2.5 volumes of ethanol. The poly(A) RNA pellet was dried and resuspended in 1 mM Tris-HCl pH 7.5, 0.1 mM EDTA.

### Decapping and recapping of poly(A) RNA

Poly(A) RNA was decapped and recapped according to methods previously described (Wulf et al. 2019). In brief, decapping of 1.5–6 µg poly(A) RNA was performed with 1.5 µL yDcps (NEB, #M0463) in 1× yDcpS reaction buffer (10 mM Bis-Tris-HCl pH 6.5, 1 mM EDTA) in 50 µL total volume for 1 h at 37°C. The decapped RNA was purified using RNA Clean & Concentrator (Zymo Research, #R1013) with the standard protocol (>17 nt recovery) and eluted in 30 µL of RNase-free water. Recapping the 5′ end of the decapped poly(A) RNA was performed with 6 µL *Vaccinia* Capping Enzyme (VCE) (NEB, #M2080) in 1× VCE reaction buffer (50 mM Tris HCl, 5 mM KCl, 1 mM MgCl_2_, 1 mM DTT, pH 8), 6 µL *Escherichia coli* inorganic pyrophosphatase (NEB, #M0361), 0.5 mM 3′-azido-ddGTP (Trilink, #N-4008), 0.2 mM *S*-adenosylmethionine (SAM) (NEB, #B9003) in 60 µL total volume for 30 min at 37°C. The recapped RNA was purified with RNA Clean & Concentrator as above.

### Adaptation of recapped poly(A) RNA

Azido-ddGTP recapped RNA (1–2 µg) was concentrated briefly on a SpeedVac vacuum concentrator (Savant) to reduce the volume to ∼5–10 µL. Copper-free Click Chemistry reactions were performed in a total volume of 50 μL, containing 25% v/v PEG 8000 (NEB, #B1004) and 20% v/v acetonitrile (Sigma-Aldrich, #271004) in 0.1 M sodium acetate buffer, pH 4 (10×, Alfa Aesar, #J60104) and 10 mM EDTA (50×, Invitrogen, #15575-038). Azido-ddGTP recapped RNA and the 3′-DBCO RNA adapter (200 nmol, final concentration of 4 µM) were added and shaken for 2 h at room temperature. Then, acetonitrile was removed by brief concentration on a SpeedVac, and the adapted RNA recovered using RNA Clean & Concentrator (Zymo Research, #R1013) following the protocol to separate large RNA (desired) from small RNA (excess adapter).

### Validation of an unannotated ADGRE1 isoform

cDNA for 5′ RACE sequencing was made with the 5′ RACE protocol using the template-switching RT Enzyme Mix (NEB, #M0466). ADGRE1 cDNA was reverse transcribed from total GM12878 RNA using a template-switching oligo (TSO) (GCTAATCATTGCAAGCAGTGGTATCAACGCAGAGTACATrGrGrG) and a poly(dT) reverse transcription primer. ADGRE1 cDNA was PCR amplified using a forward primer (underlined sequence of the TSO) and a gene-specific reverse primer with Q5 Hot Start High-Fidelity 2× Master Mix (NEB, #M0494S). cDNA was prepared for sequencing using the barcoded NBD 104 expansion of the SQK-LSK109 protocol following the manufacturer's recommendations and sequenced using a Flongle flow cell. Ionic current traces were base called with MinKnow real-time base calling using the high-accuracy model.

### MinION RNA sequencing

Poly(A) RNA samples were split and either processed for cap-adaptation (treated) or used as a matched negative control (untreated). Both treated and untreated poly(A) RNA (500–775 ng) were prepared for nanopore direct RNA sequencing generally following the ONT SQK-RNA002 Kit protocol, including the optional reverse transcription step recommended by ONT. Instead of using Superscript III, as in the ONT protocol, Superscript IV (Thermo Fisher, #18091050) was used for reverse transcription. RNA sequencing on the MinION was performed using ONT R9.4 flow cells and the standard MinKNOW protocol (48 h sequencing script) as recommended by ONT, with one exception. We collected bulk phase continuous data files for 2 h of sequencing and then restarted the sequencing runs after the 2 h of initial sequencing.

### Base calling, filtering, and alignments

We used the ONT Guppy workflow (version 3.0.3 + 7e7b7d0 configuration file rna_r9.4.1_70bps_hac.cfg) for base calling direct RNA. NanoFilt (version 2.5.0) (De Coster et al. 2018) was used to classify reads as pass if the preread average Phred-score threshold was greater than or equal to 7 and fail if less than 7. A custom python script was used to convert the U's in the guppy base called sequence to T's. Porechop (version 0.2.4) was used to identify the 5′ adapter sequence (https://github.com/rrwick/Porechop). We used barcode_diff 1 and barcode_threshold 70 or 74 for S288C or GM12878 reads, respectively (Supplemental Fig. 1). The adapters were untrimmed while optimizing parameters and trimmed for all other analysis. The barcode search sequence, TCCCTACACGACGCTCTTCCGA, was added to the end of the adapter list in the adapters.py. Reads were then aligned to the appropriate reference, sacCer3 or GRCh38, using minimap2 (Li 2018) (version 2.16-r922) with recommended conditions.

### TSS filtering pipeline

TSS filtering pipeline is available on GitHub (https://github.com/mitenjain/dRNA_capping_analysis). Reads were mapped to the human genome (GRCh38.p3.genome.fa) using Minimap2 (version 2-2.9) with the following parameters: –secondary = no -ax splice -k14 -uf. Remaining secondary alignments were removed (using samflag -F 2048). Reads containing 15 or more soft or hard clipped bases at the 5′ end of the read were removed to avoid positioning TSS at the wrong locations (properly mapped reads). Finally, for adapted reads only, the untrimmed reads containing less than 15 soft or hard clipped bases were flagged and the equivalent trimmed reads removed. This filter removes the reads that were called adapted by Porechop but the adapter is matching the genomic sequence (true positive reads). Unless otherwise stated, subsequent analyses for the untreated sample or unadapted fraction of the treated sample were done using the properly mapped reads. For the adapted fraction of the treated sample, subsequent analysis was done on the properly mapped/true positive reads.

### Identification of novel 5′ ends

In order to identify TSS that have not previously been annotated, only the 5′ end reads mapping to 300 or more bases away from GENCODE v32 (Frankish et al. 2019) annotated TSSs were retained. Next, we used reads that aligned to GENCODE genes. Default parameters were used to filter the GM12787 data. GENCODE v32 (Frankish et al. 2019) was used as our known isoform and start site annotation.

### 5′ RACE candidate gene selection

RACE candidates were selected from the unannotated genes identified by the TSS filtering pipeline. Genes with a cap-adapted read which had a TSS at unannotated exons or internal exons were considered as candidates. Transcripts with a longer or shorter annotated first exon were excluded from this analysis. Eighty-eight candidate genes were selected for 5′ RACE validation.

### Mapping 5′ end to chromatin marks, ChIP-seq, and CAGE data

Encode data were downloaded using the following accession numbers: ENCFF340BYJ (POLR2 ChIP-seq), ENCFF289XSX (SPI1 ChIP-seq), ENCFF093VXI (DNase-seq), and ENCFF580WIH (CAGE). For plotting purposes, all the data sets were down sampled to the total number of read defining novel TSS in the treated sample (9116 reads). To avoid repeats in the data set, all the reads within a 50 bp window were merged. The most 5′ end of the mapped reads were used to define the reference locations. deepTools 3.3.0 (Ramírez et al. 2016) was used to perform the heatmap plotting with the following parameters: computeMatrix reference-point -a 1000 -b 1000 and plotHeatmap –missingDataColor = #440154FF –colorList “#440154FF,#238A8DFF,#FDE725FF” “#440154FF,#238A8DFF,#FDE725FF” “#440154FF,#238A8DFF,#FDE725FF.”

### Annotation of the nanopore reads

The genomic position of the 5′ end of mapped nanopore reads was compared to GENCODE annotation v32 (Frankish et al. 2019) using bedtools (v2.27.1) with the following parameters: bedtools closest -t first -D a -iu -s. For each matching annotation, the gene_type information was used to quantify the overlap with a specific gene type (for example “protein-coding”). The quantification was normalized to 1 and plotted in a bar plot with colors representing the gene_type.

### StringTie2 and FLAIR

StringTie2 (v2.1.5) was executed using the following command: stringtie -L -o untreated_reads_stringtie.gff untreated_reads.bam.

The basic FLAIR (commit 150c33e915aaf72ad3d39bca0a99d969850a284c) workflow was followed using these commands:

First, python flair/bin/bam2Bed12.py -i untreated_reads.bam > untreated_reads.bed12.
Second, python flair.py correct -q untreated_reads.bed12 -c GRCh38_with_decoys.fa.sizes -f gencode.v32.annotation.gtf.
Third, python flair.py collapse -g GRCh38_with_decoys.fa -r untreated_reads.fq -q unttreated_reads.bed12.

### Ionic current visualization

The ionic current traces were visualized using a custom MATLAB script. Determining the ionic current of the cap-adapter is described in Supplemental Methods and visualized in Supplemental Figure 10.

## DATA DEPOSITION

Data and analysis scripts can be found at the following GitHub: https://github.com/mitenjain/dRNA_capping_analysis. Additionally, the nanopore data can be found at ENA with the accession number PRJEB43374.

## SUPPLEMENTAL MATERIAL

Supplemental material is available for this article.

## COMPETING INTEREST STATEMENT

M.G.W., I.S., G.T., J.B., I.R.C., and L.E. are employees of New England Biolabs Inc. New England Biolabs commercializes reagents for molecular biology applications. M.A. holds options in Oxford Nanopore Technologies (ONT). M.A. is a paid consultant to ONT. L.M., M.A., and M.J. received reimbursement for travel, accommodation, and conference fees to speak at events organized by ONT. M.A. is an inventor on 11 UC patents licensed to ONT (6,267,872, 6,465,193, 6,746,594, 6,936,433, 7,060,50, 8,500,982, 8,679,747, 9,481,908, 9,797,013, 10,059,988, and 10,081,835). M.A. received research funding from ONT.

## Supplementary Material

Supplemental Material

## Figures and Tables

**Logan Mulroney:**
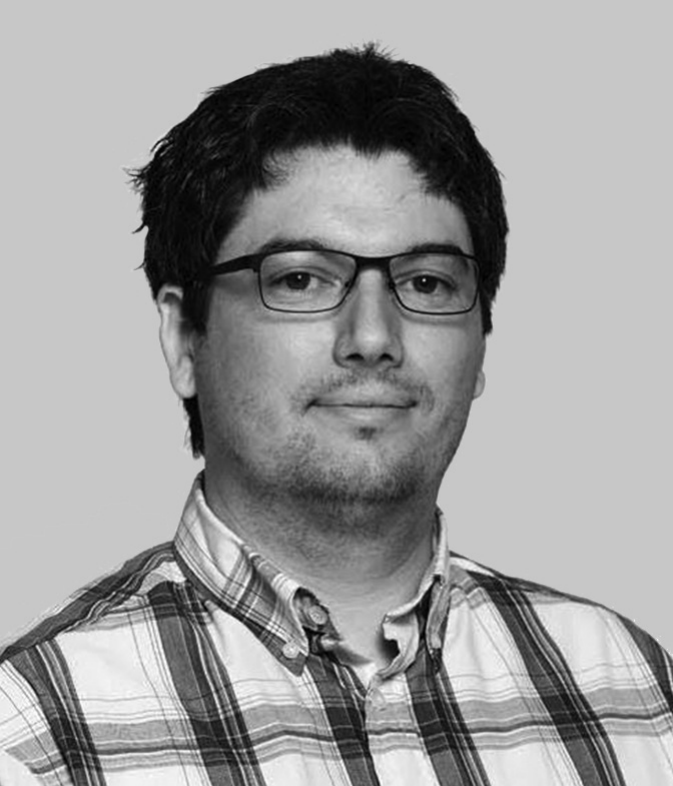


**Madalee Wulf:**
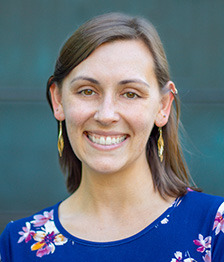


## MEET THE FIRST AUTHORS

***Meet the First Author(s)* is a new editorial feature within *RNA*, in which the first author(s) of research-based papers in each issue have the opportunity to introduce themselves and their work to readers of *RNA* and the RNA research community. Logan Mulroney and Madalee Wulf are the co-first authors of this paper, “Identification of high-confidence human poly(A) RNA isoform scaffolds using nanopore sequencing.” Logan did this work as a graduate student at the University of California Santa Cruz in Mark Akeson's laboratory, working on nanopore sequencing methods, and is currently a postdoctoral fellow at the EMBL-European Bioinformatics Institute and the Center for Genomic Sciences at the Italian Institute of Technology in the laboratories of Ewan Birney and Tommaso Leonardi. Madalee is a Research Scientist in the RNA Division at New England Biolabs in the laboratory of Ivan Correa, where she develops chemical tools for the detection and sequencing of RNA.**

**What are the major results described in your paper and how do they impact this branch of the field?**

Sequencing individual RNA molecules from their 3′ end through their 5′ end provides a level of information not easily achieved by RNA-seq or mass spectrometry. The major results of this work were improving confidence in detecting native 5′ ends of individual RNA molecules. This enabled us to identify both annotated and unannotated transcription start sites (TSSs) from direct RNA nanopore data using a method we are calling Nanopore ReCappable sequencing (NRCeq). Further, this work represents an important milestone for experimental evidence of TSS detection from direct RNA nanopore data, which complements current predictive algorithms nicely.

**What led you to study RNA or this aspect of RNA science?**

**LM:** My interest in RNA started when I joined Professor Manuel Ares's group at UC Santa Cruz as an undergraduate, trying to understand transcript competition for the splicing machinery. During my PhD I got interested in protein, DNA, and RNA modifications and how to detect them using nanopores. The collaboration with NEB was an excellent opportunity to learn more about the RNA m^7^G cap and how we can exploit the cap to improve nanopore sequencing.

**MW:** Since I joined New England Biolabs over five years ago, my projects have primarily focused on different aspects of RNA sequencing and RNA modification detection. This project was my first introduction into nanopore sequencing technologies and offered a wonderful opportunity to dive deep into a specific problem relating to TSS detection. Our expertise in nucleic acid chemistry and enzymes allowed the team at NEB to come up with a unique chemo-enzymatic strategy that ultimately allowed the detection of full-length RNA scaffolds, many of which are currently unannotated. I look forward to working on more nanopore sequencing projects in the future.

**During the course of these experiments, were there any surprising results or particular difficulties that altered your thinking and subsequent focus?**

It was surprisingly difficult to acquire full-length direct RNA nanopore reads, which we thought would be much easier. Consequently, we completely redesigned the entire protocol multiple times until we developed a protocol that produced a satisfactory proportion of cap-adapted molecules that we could sufficiently analyze the transcriptome. Both teams believed in the importance of this project and its ability to work, so we all persisted through challenging experimental results to ultimately create a technique that is usable by others in the research community.

**Are there specific individuals or groups who have influenced your philosophy or approach to science?**

**LM:** One of the many people that influenced my approach to science was my PhD advisor, Professor Mark Akeson. He taught me that an experiment should be designed such that you learn something regardless of the result and to evaluate a phenomenon using multiple approaches to eliminate competing theories. These two aspects of my approach to science help me be more critical of my own results and confident in my conclusions about my data.

**MW:** Professor Ming Hammond was my first research advisor as an undergraduate at UC Berkeley, and she absolutely influenced my approach to science. She taught me the importance of scientific integrity and time management—two aspects that are critical to my scientific process today. She also challenged me to think critically about my research and to put my colleagues’ experiments above my own. It's an important lesson on scientific respect that I was eager to learn early on in my career. I cannot thank her enough for the impact she had on me as a young female scientist.

**What were the strongest aspects of your collaboration as co-first authors?**

Logistics, communication, and perseverance. The logistics of actually performing these experiments took a remarkable level of coordination: RNA was isolated at UCSC, shipped to NEB for the chemical adaptation portion of the protocol, and then shipped back to UCSC for sequencing. Furthermore, both groups had bioinformatics expertise, requiring coordination for our analysis pipelines, particularly as software needed to be developed to analyze our data.

During this collaboration, our groups met at least twice a month to discuss results of each experiment. Maintaining that level of open and honest communication allowed us to each apply our specialties to overcome complications from all of the experiments. There was no portion of the project that was isolated to one of the two groups. We all contributed feedback and ideas for each aspect of the collaboration: enzymatic and chemical processing; the adapter design; sequencing protocols; alternative explanations for results; and the analysis pipeline. The project as a whole benefited from every aspect shared by all collaborators.

For much of the project, we were organizing meetings in three different time zones—a real testament of the dedication and perseverance we all committed to this project. Needless to say, we were well-prepared for online meetings during the pandemic and did not miss a beat transitioning to remote-only work.

Being equals in our co-first author status really highlighted the strengths of our collaboration and allowed us to see this project to completion.
