# Supplementary material for: Identification of high-confidence human poly(A) RNA isoform scaffolds using nanopore sequencing
Source: RNA. 2022 Feb;28(2):162–76. doi: 10.1261/rna.078703.121 (PMC8906549; doi:10.1261/rna.078703.121)
Supplement: Supplemental Material [file supp_28_2_162__DC1.html]

Identification of high-confidence human poly(A) RNA isoform scaffolds using nanopore sequencing — Supplemental Material 

# Identification of high-confidence human poly(A) RNA isoform scaffolds using nanopore sequencing

## Supplemental Material

- Supplemental\_Figure\_S1.pdf
- Supplemental\_Figure\_S2.pdf
- Supplemental\_Figure\_S3.pdf
- Supplemental\_Figure\_S4.pdf
- Supplemental\_Figure\_S5.pdf
- Supplemental\_Figure\_S6.pdf
- Supplemental\_Figure\_S7.pdf
- Supplemental\_Figure\_S8.pdf
- Supplemental\_Figure\_S9.pdf
- Supplemental\_Figure\_S10.pdf
- Supplemental\_Figure\_S11.pdf
- Supplemental\_Methods.pdf
- Supplemental\_Results.pdf
- Supplemental\_Sequences.pdf
- Supplemental\_Table\_S1.pdf
- Supplemental\_Table\_S2.pdf
- Supplemental\_Table\_S3.pdf
- Supplemental\_Table\_S4.pdf
- Supplemental\_Table\_S5.pdf
- Supplemental\_Table\_S6.pdf
- Supplemental\_Table\_S7.pdf
