# Supplementary material for: Identification of high-confidence human poly(A) RNA isoform scaffolds using nanopore sequencing
Source: RNA. 2022 Feb;28(2):162–76. doi: 10.1261/rna.078703.121 (PMC8906549; doi:10.1261/rna.078703.121)
Supplement: Supplemental Material [file supp_078703.121_Supplemental_Figure_S1.pdf]

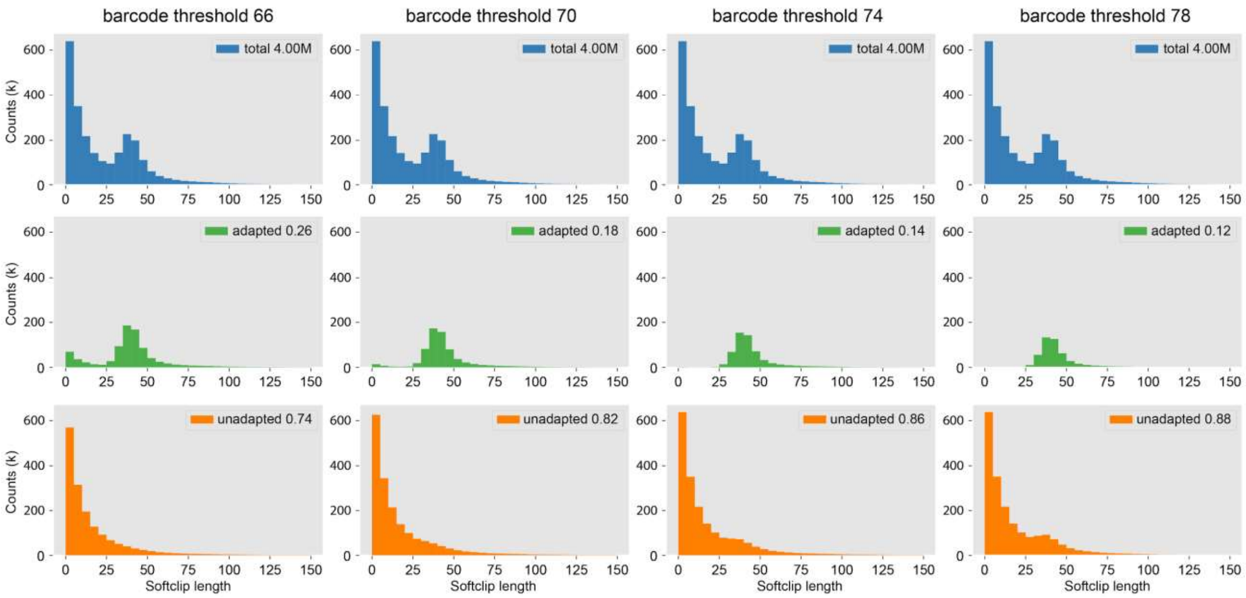

Supplementary Figure 1 GM12878 Porechop parameter optimization.
The 5’ adapter is a 45-nucleotide nt oligomer and is not expected to align anywhere in the human genome, thus it will get softclipped or hardclipped in the alignments. Adapted reads should have a soft or hardclip ~40 nt on the 5’ end. Histograms of the 5’ softclip or hardclip lengths from untrimmed sequences shows the adapted and unadapted reads. Each column is a Porechop barcode threshold cut off. The top row (blue) are the softclip and hardclip lengths from all the reads. The legend shows the number of reads. The middle row (green) shows the softclip and hardclip lengths from reads identified by Porechop as adapted. The legend is the proportion of cap-adapted reads. The bottom row (orange) are the reads Porechop could not identify the adapter in. We pick a threshold cut off such that the false positives (adapted softclip near 0) and the false negatives (unadapted softclip ~40) are minimized. For GM12878, 74 was the optimal barcode threshold.
