# Supplementary material for: Identification of high-confidence human poly(A) RNA isoform scaffolds using nanopore sequencing
Source: RNA. 2022 Feb;28(2):162–76. doi: 10.1261/rna.078703.121 (PMC8906549; doi:10.1261/rna.078703.121)
Supplement: Supplemental Material [file supp_078703.121_Supplemental_Figure_S2.pdf]

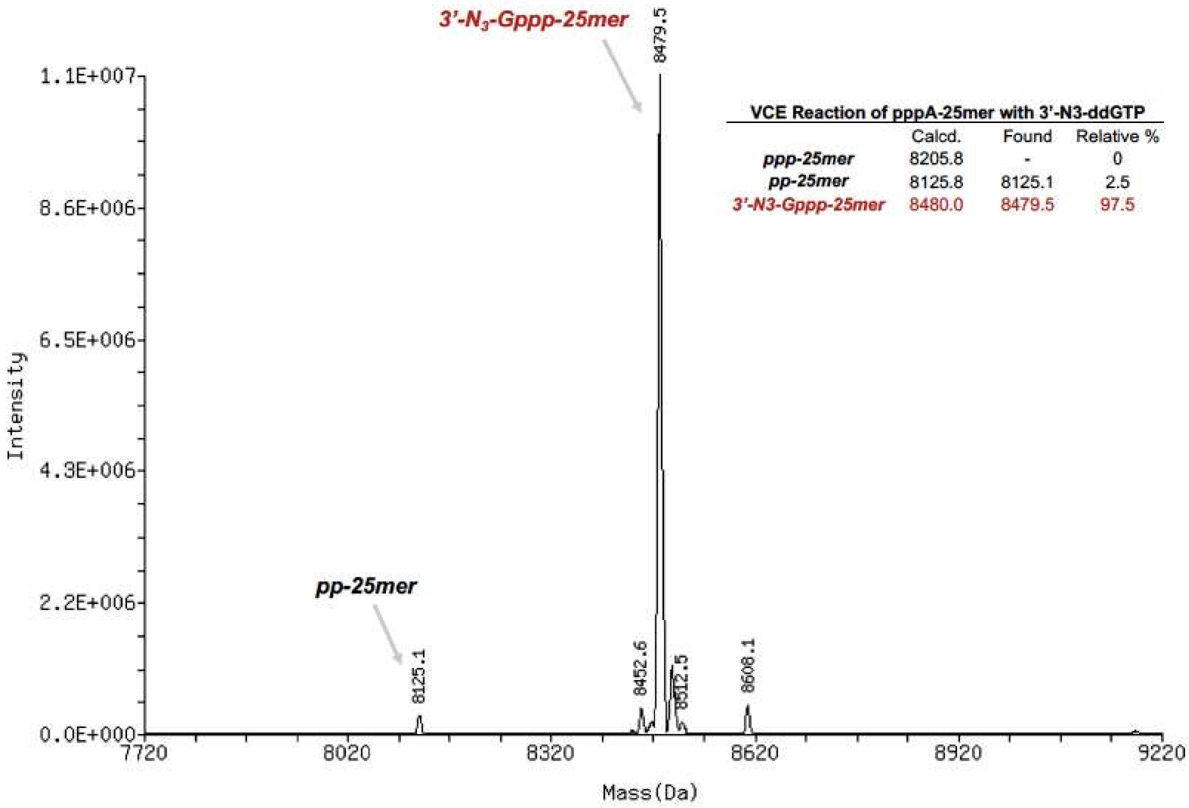

Supplementary Figure 2 *Vaccinia* Capping Enzyme caps RNA with 3'-azido-ddGTP. Deconvoluted ESI-MS spectra of a synthetic 25-nucleotide 5′-triphosphate RNA oligomer (ppp-25mer) capped with 3'-azido-ddGTP. Tandem Liquid Chromatography-Mass Spectrometry (LC-MS/MS) was performed on a Vanquish Horizon UHPLC System coupled with a Thermo Q-Exactive Plus mass spectrometer operating under negative electrospray ionization mode (–ESI). MS data acquisition was performed in the scan mode. ESI-MS raw data was deconvoluted using Promass HR (Novatia). The composition of each peak was determined by comparison with calculated average atomic mass. The results show nearly complete oligomer capping after 60 min incubation with VCE (see Methods for capping conditions).
