# Supplementary material for: Identification of high-confidence human poly(A) RNA isoform scaffolds using nanopore sequencing
Source: RNA. 2022 Feb;28(2):162–76. doi: 10.1261/rna.078703.121 (PMC8906549; doi:10.1261/rna.078703.121)
Supplement: Supplemental Material [file supp_078703.121_Supplemental_Figure_S3.pdf]

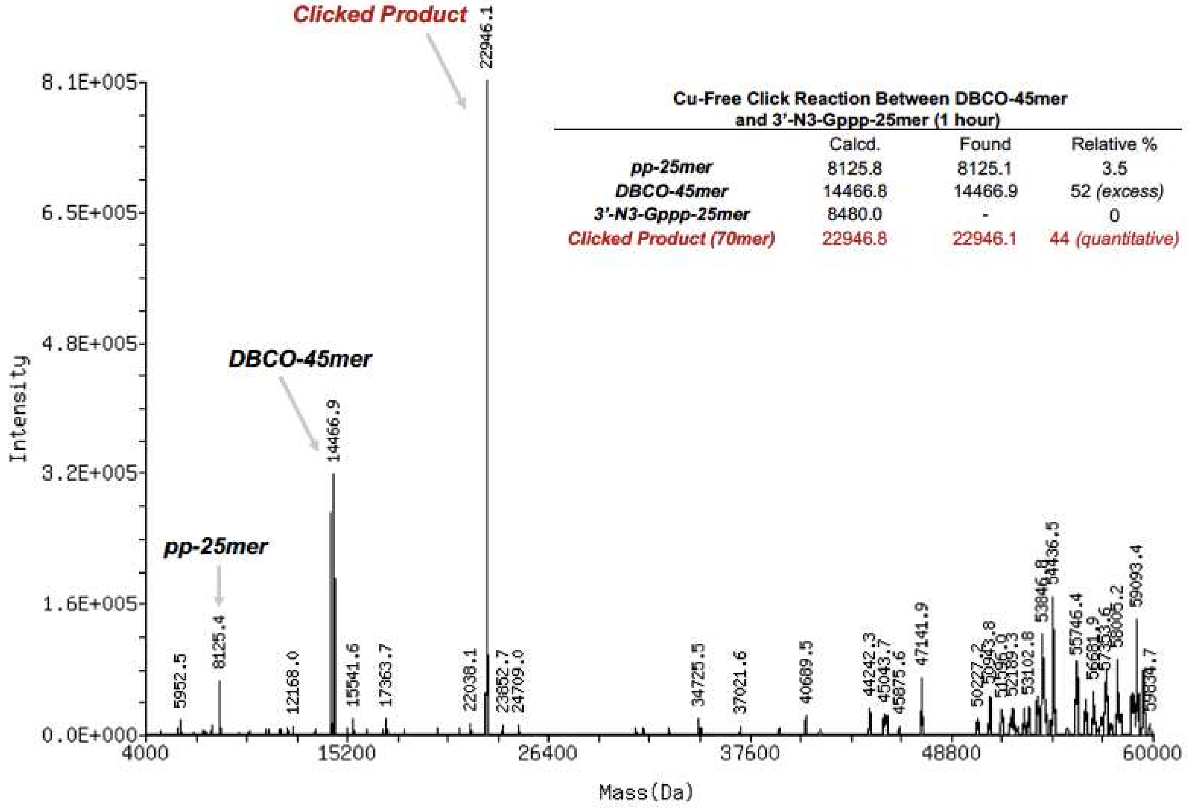

Supplementary Figure 3 Copper-free Click Chemistry enables attachment of an oligonucleotide adapter to azido-ddGTP recapped RNA. Deconvoluted ESI-MS spectra of the 25-nucleotide azido-ddGTP capped RNA oligomer from Supplementary Figure 2 coupled with the 3′-DBCO RNA adapter (DBCO-45mer). LC-MS/MS and spectral deconvolution were performed as described in Supplementary Figure 2. The composition of each peak was determined by comparison with calculated average atomic mass. The results show the azido-ddGTP capped RNA is entirely consumed after 60 min forming the desired adapted RNA (“clicked” product). Excess of unreacted 3′-DBCO adapter, and some carryover 5’-diphosphate RNA (pp-25mer) from the previous reaction, were also detected.
