# Supplementary material for: Identification of high-confidence human poly(A) RNA isoform scaffolds using nanopore sequencing
Source: RNA. 2022 Feb;28(2):162–76. doi: 10.1261/rna.078703.121 (PMC8906549; doi:10.1261/rna.078703.121)
Supplement: Supplemental Material [file supp_078703.121_Supplemental_Figure_S4.pdf]

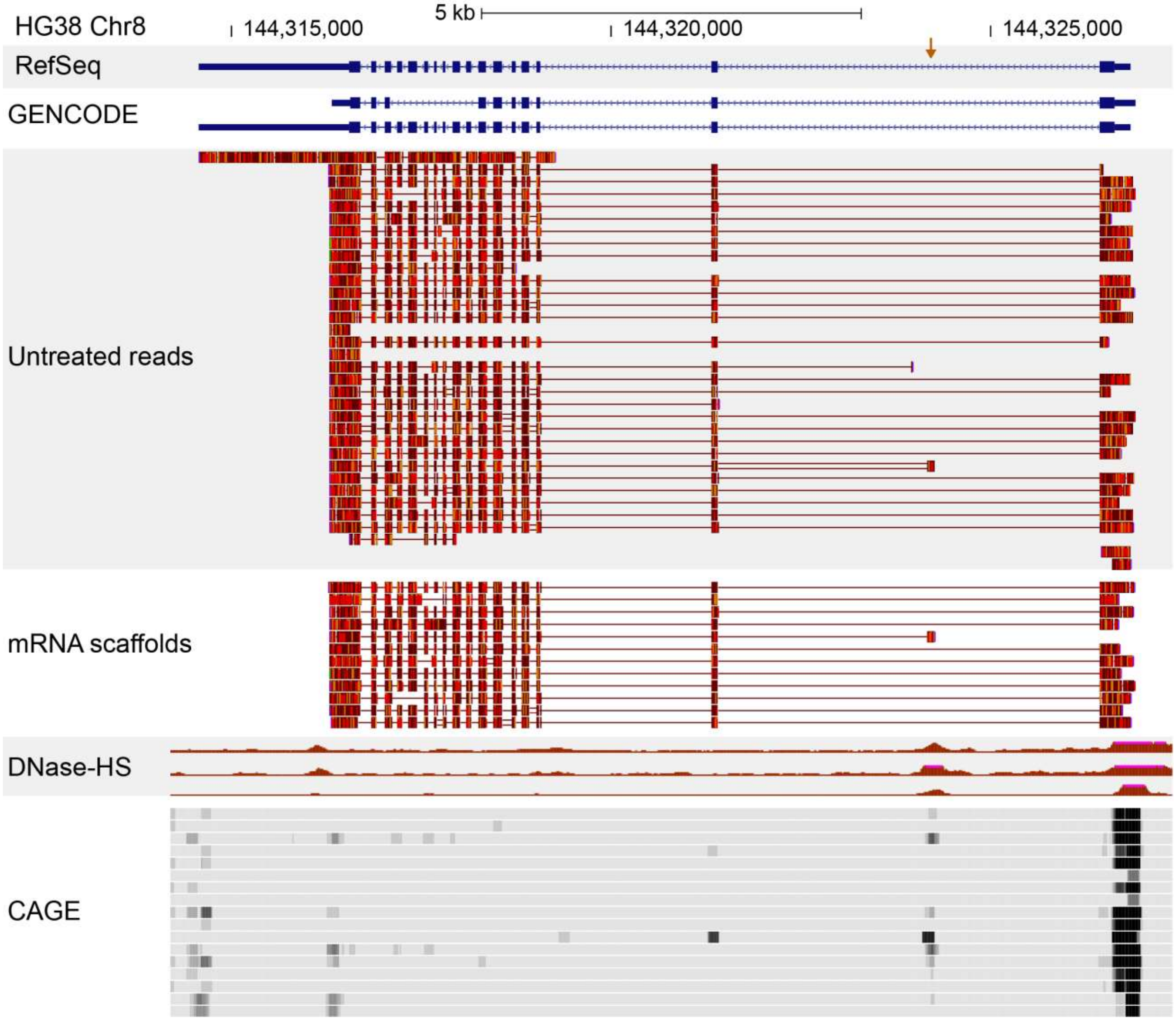

Supplementary Figure 4 Evidence for an unannotated Diacylglycerol O-Acyltransferase 1 (DGAT1) isoform is supported by a single high-confidence mRNA scaffold. The row entitled mRNA scaffolds includes 12 aligned reads in the 3′-to-5′ orientation. Most of these aligned to a GENCODE v.32 annotated isoform. One of these mRNA scaffolds (orange arrow) corresponds to an unannotated first exon of a proposed unannotated DGAT1 isoform. This unannotated isoform is also observed among the untreated reads. However, untreated reads lack strong evidence of a mature mRNA 5′ end because they are not cap-adapted. The first exon of the proposed unannotated isoform is consistent with open chromatin revealed by the DNase-HS data.
