# Supplementary material for: Identification of high-confidence human poly(A) RNA isoform scaffolds using nanopore sequencing
Source: RNA. 2022 Feb;28(2):162–76. doi: 10.1261/rna.078703.121 (PMC8906549; doi:10.1261/rna.078703.121)
Supplement: Supplemental Material [file supp_078703.121_Supplemental_Figure_S5.pdf]

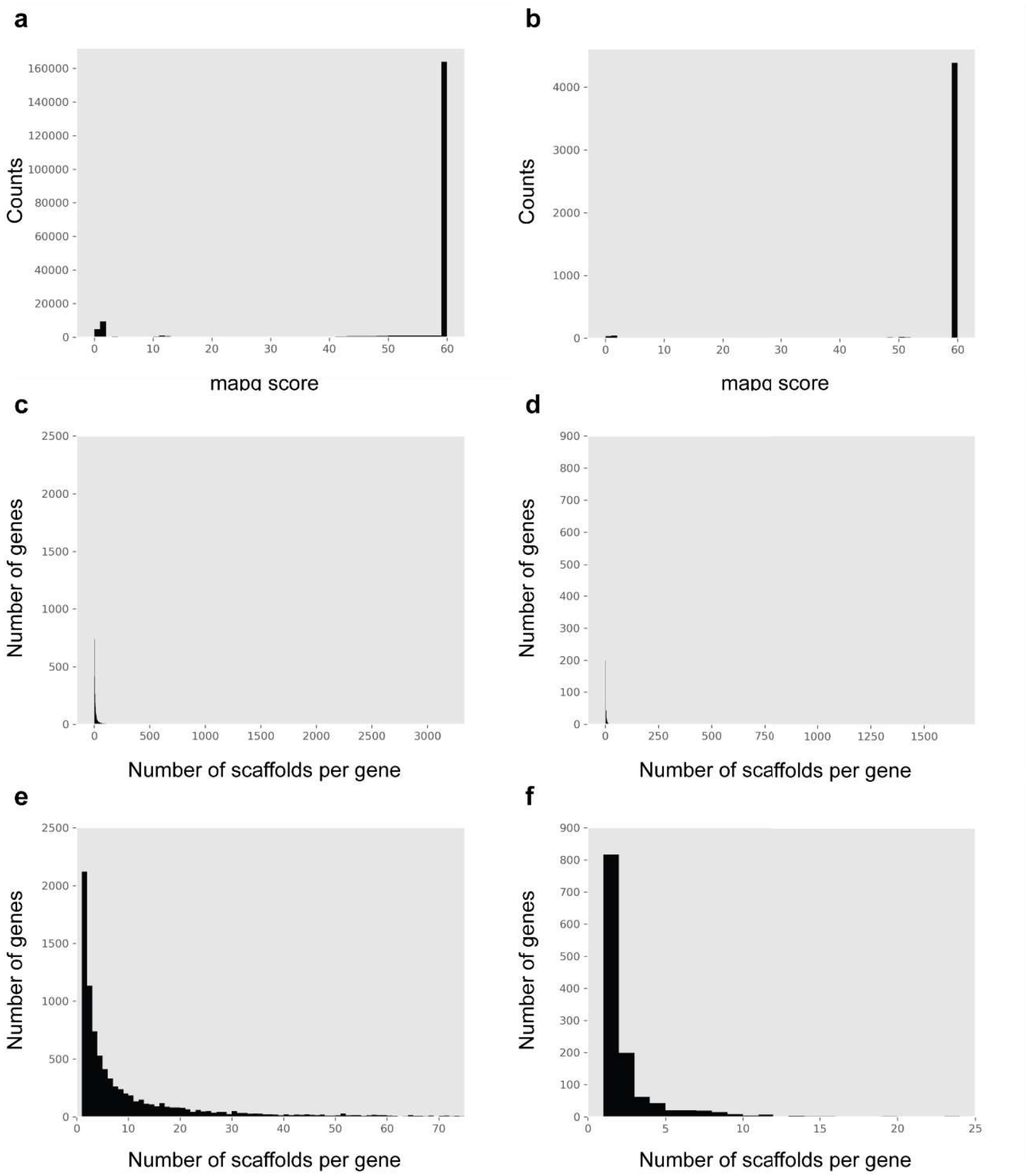

Supplementary Figure 5 RNA isoform scaffold summary. (**a**) A histogram of map quality (mapq) scores for all the full-length RNA scaffolds. Mapq scores can range from 0-to-60. The x-axis is the mapq score for each read, and the y-axis is the number of reads with the corresponding mapq score. (**b**) A histogram of mapq scores for the unannotated RNA isoform scaffolds. The axes are the same as in panel **a**. (**c**) A histogram of the number of scaffolds per gene for the full-length RNA scaffolds. The x-axis is the number of scaffolds that aligned to each gene and the y-axis is the number of genes that had the corresponding number of scaffolds. (**d**) A histogram of the number of scaffolds per gene for the unannotated full-length RNA scaffolds. The axes are the same as in panel **c**. (**e**) The same plot as in panel **c**, except the x-axis has been narrowed to view most of the data. (**f**) The same plot as in panel **d**, except the x-axis has been narrowed to view most of the data.
