# Supplementary material for: Identification of high-confidence human poly(A) RNA isoform scaffolds using nanopore sequencing
Source: RNA. 2022 Feb;28(2):162–76. doi: 10.1261/rna.078703.121 (PMC8906549; doi:10.1261/rna.078703.121)
Supplement: Supplemental Material [file supp_078703.121_Supplemental_Figure_S6.pdf]

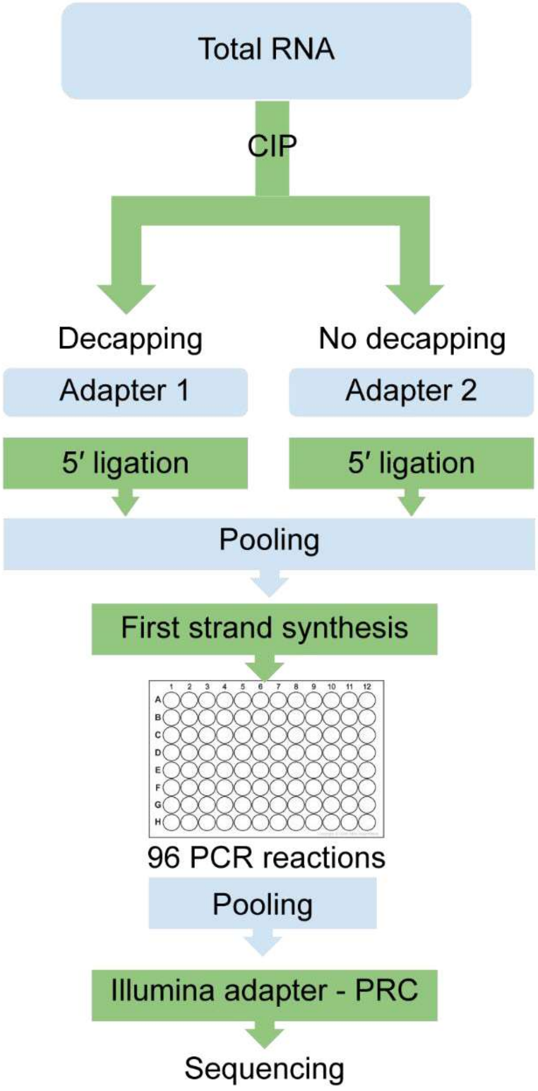

Supplementary Figure 6 Experimental design for RACE validation of 93 isoforms from 88 genes. A flow chart diagraming how the multiplexed RACE experiment was conducted with controls.
