# Supplementary material for: Identification of high-confidence human poly(A) RNA isoform scaffolds using nanopore sequencing
Source: RNA. 2022 Feb;28(2):162–76. doi: 10.1261/rna.078703.121 (PMC8906549; doi:10.1261/rna.078703.121)
Supplement: Supplemental Material [file supp_078703.121_Supplemental_Figure_S8.pdf]

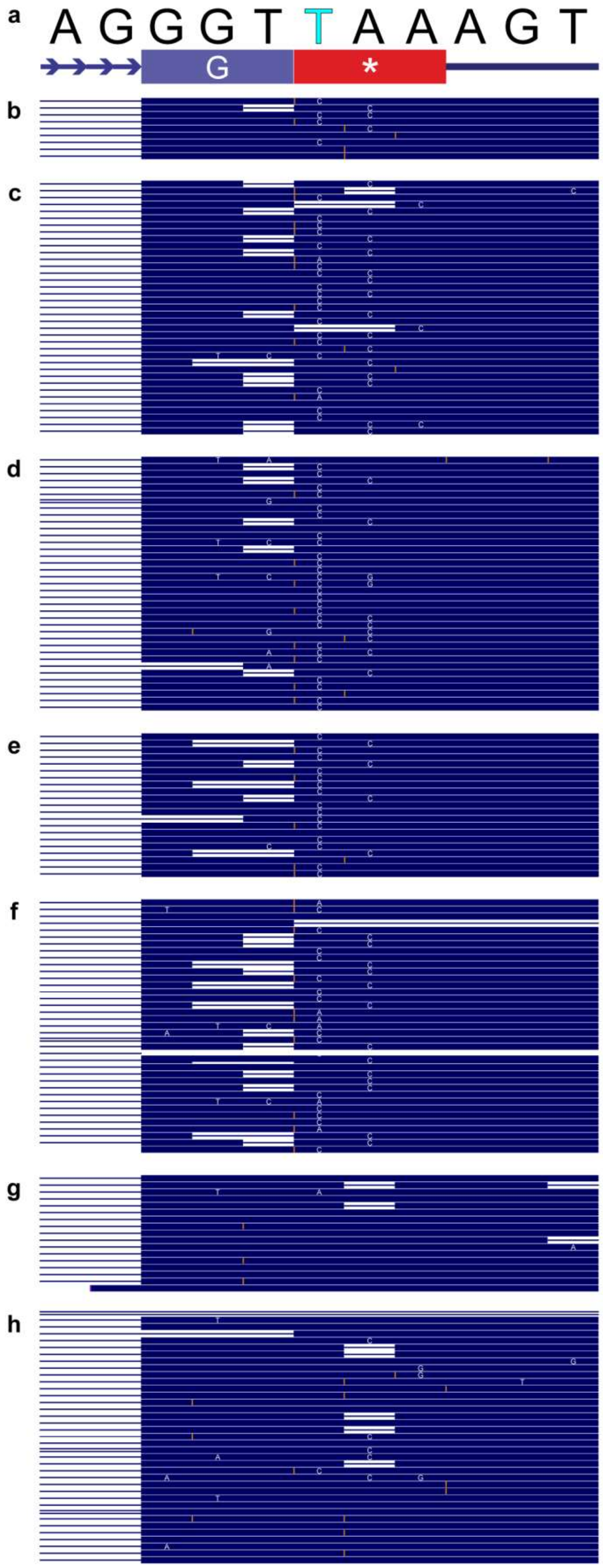

Supplementary Figure 8 Nanopore evidence for pseudouridine in the stop codon of ADGRE1 mRNA. (**a**) HG38 chr19:6,940,022-6,940,032 which corresponds to eleven nucleotides in the last exon of ADGRE1. The G (blue background) in the second row is a glycine of the ADGRE1 gene product. The * (red background) is the canonical stop codon for ADGRE1. A pseudouridine at the first nucleotide of that stop codon can promote ribosome read through in other genes[36]. (**b**) High confidence mRNA scaffolds aligned to the unannotated isoform. (**c**) Treated-sample reads aligned to the unannotated isoform. (**d**) Untreated-sample reads aligned to the unannotated isoform. (**e**) RNA reads corresponding to an annotated ADGRE1 isoform from a previous study[12]. (**f**) RNA reads corresponding to the unannotated ADGRE1 isoform from a previous study[12]. (**g**) In vitro transcript reads composed of canonical nucleotides[12]. (**h**) cDNA amplicon reads derived from [12]. In panels (b-to-h), dark blue is nanopore base calls that match the reference sequence. Thick white horizontal lines are nucleotide deletions in the nanopore reads. Orange vertical lines are nucleotide insertions in the nanopore reads. White letters are base calls that disagree with the reference sequence.
