# Supplementary material for: Identification of high-confidence human poly(A) RNA isoform scaffolds using nanopore sequencing
Source: RNA. 2022 Feb;28(2):162–76. doi: 10.1261/rna.078703.121 (PMC8906549; doi:10.1261/rna.078703.121)
Supplement: Supplemental Material [file supp_078703.121_Supplemental_Figure_S9.pdf]

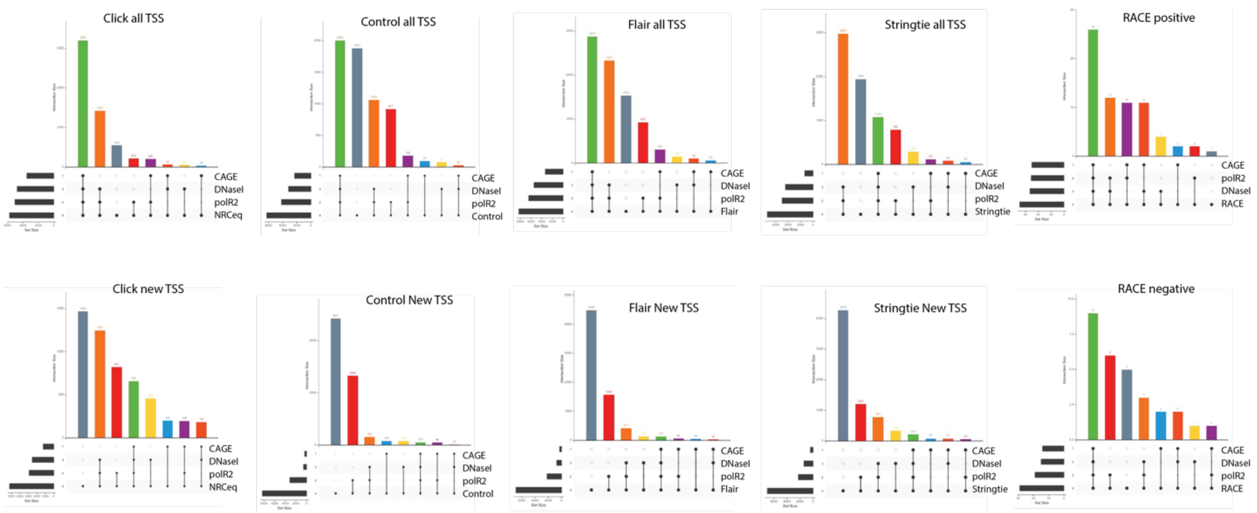

Supplementary Figure 9: Comparison of datasets TSS. The datasets used to compare with known genomic marks associated with TSS are : [1] click TSS, [2] Control TSS, [3] RACE TSS, [4] Flair predictions and [5] Stringtie predictions. For each dataset, the position matching the most 5′ end aligned to GR38 is extracted. For each datasets (excluding the RACE TSS), a subset of TSS corresponding to the new TSS are extracted. New TSS are defined as TSS that is located 300 bp away from any annotated gene start (gencode) . All datasets (excluding RACE TSS) are downsampled to the same number of entries and merged. Orthogonal markers of TSS (CAGE, Pol2 and DNase) were downloaded from Encode using the following accession numbers (Duplicate CAGE: ENCFF103DEY, ENCFF260HMC; DNaseI-seq: ENCFF235KUD and ENCFF491BOT and Pol2 ChIP: ENCFF303DQB and ENCFF324NBP). Duplicates were merged using bedtools (bedtools merge -s -c 6 -o distinct -i) and compared with our datasets. Multi-dataset comparison were done using bedtools multiinter (-cluster). Each colored vertical bar is the number of reads that correspond to each data set indicated by the dot plots. The black horizontal bars indicate the total number of reads for each individual dataset.
