# Supplementary material for: Identification of high-confidence human poly(A) RNA isoform scaffolds using nanopore sequencing
Source: RNA. 2022 Feb;28(2):162–76. doi: 10.1261/rna.078703.121 (PMC8906549; doi:10.1261/rna.078703.121)
Supplement: Supplemental Material [file supp_078703.121_Supplemental_Figure_S10.pdf]

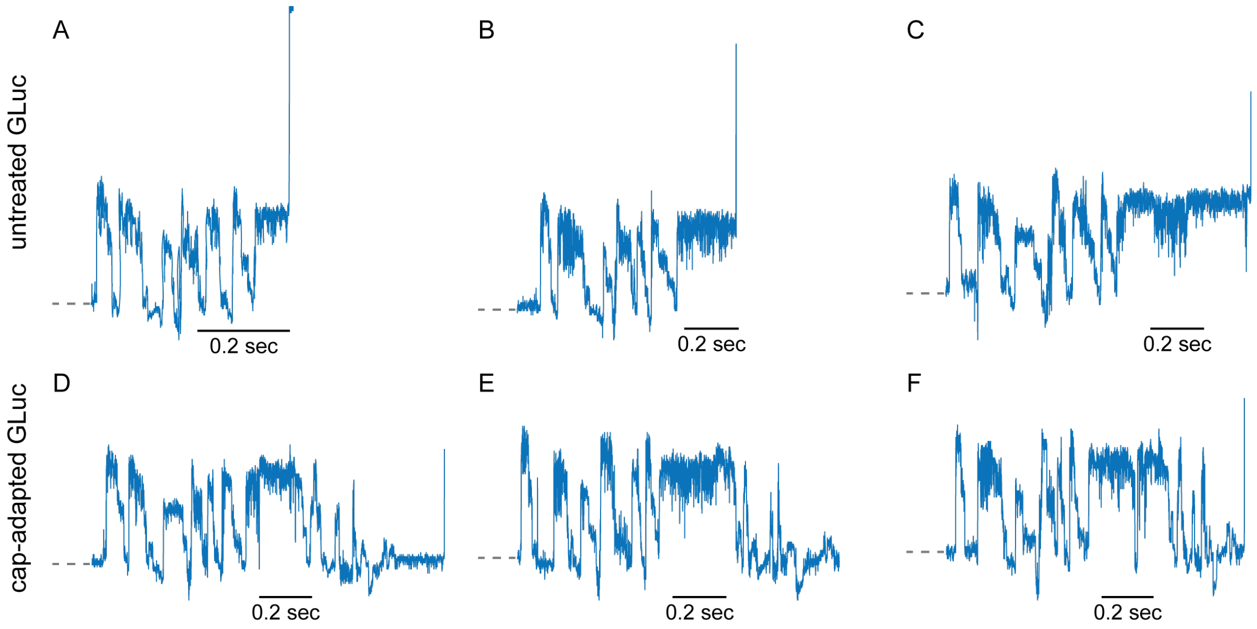

Supplementary Figure 10: Ionic current of untreated and cap-adapted GLuc 5′ ends. (**A**) An untreated *Gaussia* Luciferase (GLuc) *in vitro* transcribed (IVT) RNA ionic current trace. The ionic current has been visualized using a custom MATLAB script, and a segment of the current from the 5′ has been plotted. The black line under the ionic current trace represents 0.2 seconds, and the dashed grey line indicates where the ionic current trace was sliced and the remaining current not displayed. (**B** and **C**) Two additional ionic current traces from untreated GLuc IVT RNA. The black and dashed grey lines are the same as in panel **A**. (**D**, **E**, and **F**) Three ionic current traces from treated GLuc reads that were identified as being cap-adapted by Porechop. The black and dashed grey lines are the same as in panel **A**.
