# Supplementary material for: Identification of high-confidence human poly(A) RNA isoform scaffolds using nanopore sequencing
Source: RNA. 2022 Feb;28(2):162–76. doi: 10.1261/rna.078703.121 (PMC8906549; doi:10.1261/rna.078703.121)
Supplement: Supplemental Material [file supp_078703.121_Supplemental_Figure_S11.pdf]

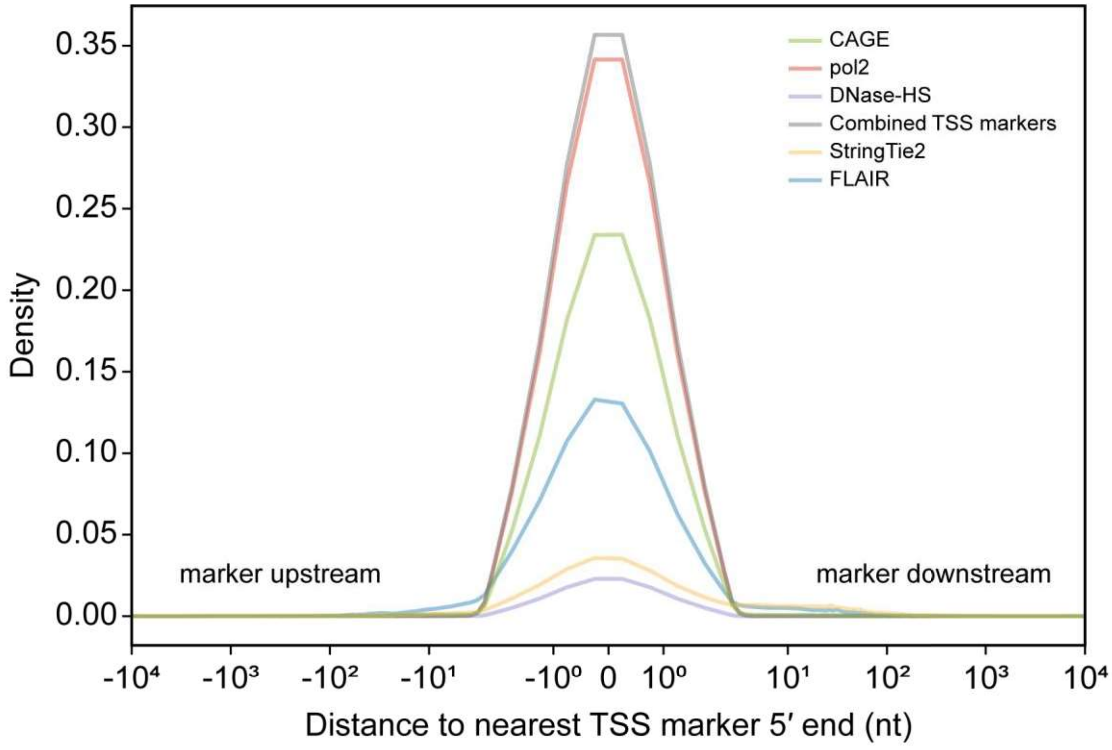

Supplementary Figure 11 Nucleotide distance of orthogonal datasets from high-confidence scaffold 5′ ends. The *x* axis is the number of nucleotides between a nanopore read 5′ end (0 position) and the closest signal from the orthogonal dataset. Negative numbers are upstream (5′) from the TSS; positive numbers are downstream (3′) from the TSS. The *y* axis is the density at a given distance of the signal from the closest high-confidence scaffold 5′ ends.
