# Supplementary material for: Identification of high-confidence human poly(A) RNA isoform scaffolds using nanopore sequencing
Source: RNA. 2022 Feb;28(2):162–76. doi: 10.1261/rna.078703.121 (PMC8906549; doi:10.1261/rna.078703.121)
Supplement: Supplemental Material [file supp_078703.121_Supplemental_Methods.pdf]

**Synthesis of 3′-Azido RNA adapter**

The 45-nucleotide 3′-azido RNA oligomer (CUCUUCCGAUCUACACUCUUUCCCUACACGACGCUCUUCCGAUCU) was synthesized on an ABI 394 DNA synthesizer (Applied Biosystems) starting with 3′-alkyne modifier Serinol CPG (BaseClick, #BCA-02) and UltraFast RNA TBDMS RNA amidites (Glen Research: Bz-A-CE #10-3003, Ac-C #10-3015, Ac-G-CE #10-3025, and U-CE #10-3030). The oligonucleotide was deprotected according to the manufacturer's protocol using ammonium hydroxide/methylamine and purified using a Glen-Pak RNA purification cartridge (Glen Research, #60-6100) followed by PAGE. The oligonucleotide was further purified by PAGE followed by a desalting step on RP-HPLC (C-8 Higgins Analytical) using 0.1 M TEAB and acetonitrile as the mobile phase. The purified oligonucleotide was converted to 3′-azido in a total volume of 889.2 µL, containing 25% v/v DMSO in 0.2 M triethylammonium acetate buffer, pH 7 as follows (unless other specified, final concentrations are given): 100 µM oligomer, 20 mM $N_3$-PEG1-$N_3$ (BroadPharm, #BP-20908) and 500 µM ascorbic acid were combined and the solution briefly degassed with nitrogen. 44.4 µL of a 10 mM Copper(II)-TBTA complex in 55% aq. DMSO (500 µM final concentration) (Lumiprobes, #21050) was added and the solution briefly degassed with nitrogen. The reaction stirred for 3 h at room temperature in absence of light. The reaction was then dissolved in 0.1 M TEAB (up to 35 mL) and purified by C8 HPLC (Higgins Analytical) using 0.1 M TEAB and acetonitrile as the mobile phase to yield the 3′-azido RNA adapter.

**Adaptation of propargyl capped RNA via Copper-catalyzed Click Chemistry**

Copper-catalyzed click chemistry reactions were performed in a total volume of 10 µL, containing 25% v/v DMSO in 0.2 M triethylammonium acetate buffer, pH 7 as follows (unless other specified, final concentrations are given): 0.5 µM propargyl capped RNA, 4 µM 3′-azido RNA adapter and 500 µM ascorbic acid were combined and the solution briefly degassed with nitrogen. 0.5 µL of a 10 mM Copper(II)-TBTA complex in 55% aq. DMSO (500 µM final concentration) (Lumiprobes, #21050) was added and the solution briefly degassed with nitrogen. The reaction shaken overnight at room temperature in absence of light. The adapted RNA was recovered using RNA Clean & Concentrator (Zymo Research, #R1013).

**Porechop adapter identification**

The search sequence TCCCTACACGACGCTCTTCCGA was added to Porechop's adapters.py file as a new 5′ barcode. The native RNA nanopore reads had the U's in the sequence replaced with T's using a python script, and then quality filtered using NanoFilt to q7. Porechop was used on the quality filtered reads with the following conditions: --barcode_diff 1 --barcode_threshold 70 (S. cerevisiae) 74 (GM12878) -i file.fastq -b outputdirectory. --untrimmed was used while optimizing the threshold parameter.

***In vitro* transcription of synthetic poly(A) GLuc RNA.**

An 809 nucleotide transcript of Gaussia luciferase was synthesized by *in vitro* transcription using HiScribe T7 Quick High Yield RNA Synthesis Kit (NEB E2050S) following the manufacturer's directions. The DNA template was synthesized using PCR from the plasmid pCMV-GLuc-2 (NEB N8081S) with the LongAmp Taq 2X Master mix (NEB, M0287S) and the following PCR primers: The forward primer incorporated the T7 promoter: 5′ –

TCGAAATTAATACGACTCACTATAGGGAGACCCAA – 3′ and the reverse primer was used to add a 3′ terminal tail of 125 A residues: 5′ – (T125)ACAGTAAGAATTATTTCTAGACACAC – 3′.

**Determination of ionic current signal associated with the cap-adapter**

Both a control and treated GLuc IVT RNA were sequenced on individual MinIONs following the same protocols as all other experiments. The cap-adapter sequence was identified in the treated reads using Porechop. The ionic current traces for the cap-adapted reads and control reads that aligned within the first 25 nucleotides of the GLuc reference sequence were visualized using a custom matlab script. The ionic current at the 5′ end of control and cap-adapted GLuc reads were compared by eye to determine the ionic current associated with the cap-adapter. The ionic current for several random yeast and GM12878 cap-adapted reads was visually inspected in the same way for the same ionic current pattern at the 5′ end of the trace as was identified in the GLuc cap-adapted trace.
