# Supplementary material for: Identification of high-confidence human poly(A) RNA isoform scaffolds using nanopore sequencing
Source: RNA. 2022 Feb;28(2):162–76. doi: 10.1261/rna.078703.121 (PMC8906549; doi:10.1261/rna.078703.121)
Supplement: Supplemental Material [file supp_078703.121_Supplemental_Results.pdf]

## Putative modification detection in Direct RNA nanopore reads

We also noted a consistent U-to-C miscall in the ADGRE1 stop codon of the high confidence scaffolds (Supplementary Fig. 8b) and in the stop codon of all other reads from this study that aligned to ADGRE1 (Supplementary Fig. 8c and d). The same pattern was observed for direct nanopore reads from a prior study that aligned to longer GENCODE annotated ADGRE1 isoforms (Supplementary Fig. 8e)[1]. This pattern is consistent with nanopore miscalls caused by the conversion of uridine to pseudouridine ($\boldsymbol{\psi}$) at U516 in E. coli 16S rRNA[2]. Base miscalls relative to canonical training data in our ADGRE1 aligned reads (Supplementary Fig. 8g) strongly suggest an unannotated pseudouridine in the stop codon, which is known to cause translation read through[3]. Importantly, this miscall was absent from *in vitro* transcribed nanopore direct RNA sequence data[1]. Further experiments would be required to confirm this putative pseudouridine.


1. Workman, R. E. *et al.* Nanopore native RNA sequencing of a human poly(A) transcriptome. *Nat. Methods* **16**, 1297–1305 (2019).
2. Smith, A. M., Jain, M., Mulroney, L., Garalde, D. R. & Akeson, M. Reading canonical and modified nucleobases in 16S ribosomal RNA using nanopore native RNA sequencing. *PLoS One* **14**, e0216709 (2019).
3. Adachi, H. & Yu, Y.-T. Pseudouridine-mediated stop codon readthrough in S. cerevisiae is sequence context–independent. *RNA* vol. 26 1247–1256 (2020).
