# Supplementary material for: Identification of high-confidence human poly(A) RNA isoform scaffolds using nanopore sequencing
Source: RNA. 2022 Feb;28(2):162–76. doi: 10.1261/rna.078703.121 (PMC8906549; doi:10.1261/rna.078703.121)
Supplement: Supplemental Material [file supp_078703.121_Supplemental_Table_S1.pdf]

Supplementary Table 1 Native RNA nanopore sequencing statistics

Organism	sample	"Click" type	pass reads	N50	adapted
S. cerevisiae	Untreated 1	None	1,391,578	531	0
S. cerevisiae	Untreated 2	None	1,755,395	1,092	0
S. cerevisiae	Untreated 3	None	3,222,192	1,034	0
S. cerevisiae	Untreated 4	None	861,210	947	0
**S. cerevisiae**	**Untreated Pooled**	**None**	**7,230,375**	**957**	**0**
S. cerevisiae	Treated 1	Cu^{2+}	2,576,122	798	0.2382
S. cerevisiae	Treated 2	Cu^{2+}	3,933,777	676	0.0889
S. cerevisiae	Treated 3	Cu^{2+}	1,848,281	722	0.1451
S. cerevisiae	Treated 4	Cu^{2+}	1,382,164	575	0.0696
S. cerevisiae	Treated 5	Cu^{2+}	600,225	435	0.0618
**S. cerevisiae**	**Treated Pooled**	**Cu^{2+}**	**10,340,569**	**692**	**0.134**
S. cerevisiae	Treated 1	Cu-Free	1,128,595	737	0.3347
S. cerevisiae	Treated 2	Cu-Free	3,799,042	755	0.4138
S. cerevisiae	Treated 3	Cu-Free	1,275,112	715	0.3407
**S. cerevisiae**	**Treated Pooled**	**Cu-Free**	**6,202,749**	**744**	**0.3844**
GM12878	Untreated 1	None	1,332,182	1,572	0
GM12879	Untreated 2	None	2,497,808	1,691	0
**GM12880**	**Untreated Pooled**	**None**	**3,829,990**	**1,615**	**0**
GM12881	Treated 1	Cu-Free	640,221	1,334	0.1106
GM12882	Treated 2	Cu-Free	919,701	1,131	0.1021
GM12883	Treated 3	Cu-Free	797,451	1,212	0.1596
GM12884	Treated 4	Cu-Free	1,706,866	1,189	0.1653
**GM12885**	**Treated Pooled**	**Cu-Free**	**4,064,239**	**1,207**	**0.1413**
