# Supplementary material for: Identification of high-confidence human poly(A) RNA isoform scaffolds using nanopore sequencing
Source: RNA. 2022 Feb;28(2):162–76. doi: 10.1261/rna.078703.121 (PMC8906549; doi:10.1261/rna.078703.121)
Supplement: Supplemental Material [file supp_078703.121_Supplemental_Table_S2.pdf]

Supplementary Table 2 Effect of copper-catalyzed and copper-free click reactions on RNA integrity and nanopore read quality. The RIN was measured from S. cerevisiae total RNA after enzyme treatment and purification for each step of the cap-adaptation process using an Agilent RNA 6000 Nano Kit (mean ± SD for n = 2 experiments). Percent cap-adapted is the percent of poly(A) RNA nanopore reads identified by Porechop as cap-adapted. The read N50 is where half of the total bases sequenced are in reads of that length or longer.

	No Treatment	yDCPS	VCE	Copper-catalyzed Click Adaptation	Copper-Free Click Adaptation
**RIN**	9.5 ± 0.1	9.4 ± 0.4	8.1 ± 0.2	6.7 ± 0.2	8.1 ± 0.7
**Percent cap-adapted**	-	-	-	13.40%	38.40%
**N50**	957 nt	-	-	692 nt	744 nt
