# Supplementary material for: Identification of high-confidence human poly(A) RNA isoform scaffolds using nanopore sequencing
Source: RNA. 2022 Feb;28(2):162–76. doi: 10.1261/rna.078703.121 (PMC8906549; doi:10.1261/rna.078703.121)
Supplement: Supplemental Material [file supp_078703.121_Supplemental_Table_S3.pdf]

**Supplementary Table 3** Counts of RNA types for untreated, unadapted, and cap-adapted reads.

| RNA type | untreated | unadapted | cap-adapted |
|---|---|---|---|
| protein coding | 988063 | 1954644 | 278718 |
| Mt tRNA | 59686 | 110205 | 6 |
| processed pseudogene | 26917 | 59970 | 7014 |
| Mt rRNA | 26325 | 39885 | 154 |
| lncRNA | 22023 | 38564 | 4642 |
| unknown | 20129 | 35120 | 2823 |
| transcribed processed pseudogene | 4011 | 9612 | 1447 |
| transcribed unprocessed pseudogene | 3035 | 6087 | 631 |
| IG V gene | 1540 | 1818 | 1087 |
| unprocessed pseudogene | 670 | 1463 | 189 |
| IG C gene | 554 | 6050 | 26 |
| polymorphic pseudogene | 317 | 905 | 426 |
| snRNA | 271 | 93 | 0 |
| miRNA | 252 | 549 | 37 |
| IG V pseudogene | 227 | 1027 | 112 |
| TR J gene | 175 | 117 | 81 |
| transcribed unitary pseudogene | 172 | 395 | 31 |
| rRNA | 157 | 91 | 2 |
| snoRNA | 143 | 104 | 0 |
| TEC | 128 | 249 | 35 |
| misc RNA | 91 | 69 | 16 |
| TR C gene | 64 | 252 | 2 |
| IG J gene | 52 | 433 | 12 |
| unitary pseudogene | 14 | 25 | 1 |
| snoRNA | 5 | 3 | 8 |
| translated processed pseudogene | 5 | 7 | 1 |
| TR V gene | 4 | 6 | 1 |
| scRNA | 3 | 2 | 0 |
| TR V pseudogene | 3 | 2 | 1 |
| translated unprocessed pseudogene | 3 | 8 | 6 |

| | | | |
|---|---|---|---|
| IG J pseudogene | 2 | 15 | 1 |
| TR D gene | 2 | 0 | 0 |
| TR J pseudogene | 2 | 0 | 0 |
| snRNA | 1 | 2 | 16 |
