# Supplementary material for: Identification of high-confidence human poly(A) RNA isoform scaffolds using nanopore sequencing
Source: RNA. 2022 Feb;28(2):162–76. doi: 10.1261/rna.078703.121 (PMC8906549; doi:10.1261/rna.078703.121)
Supplement: Supplemental Material [file supp_078703.121_Supplemental_Table_S4.pdf]

Supplementary Table 4 Nanopore Recappable-seq TSS validation by 5′ RACE. 90 genes c
(including two control genes) are listed below. 5′ RACE signal validated 64 TSS for 61 genes
indicates a low number of reads.

Gene	Chromosome	start	end	strand	RPPH reads	no RPPH reads	RPPH %
AMBRA1	chr11	46483098	46483198	-	3442	0	100
BCL2A1	chr15	80017734	80017834	-	7392	0	100
BLK	chr8	11538185	11538285	+	1115	0	100
CCDC12	chr3	46927226	46927326	-	3276	0	100
CCR10	chr17	42682390	42682490	-	147	0	100
CORO1B	chr11	67438647	67438747	-	1	0	100
CRELD2	chr22	49925093	49925193	+	2453	0	100
FBXL15	chr10	102421091	102421191	+	1625	0	100
FHL2	chr2	105373930	105374030	-	2001	0	100
FLOT2	chr17	28896992	28897092	-	1015	0	100
GEMIN7	chr19	45075638	45075738	+	31	0	100
ICA1	chr7	8134516	8134616	-	2234	0	100
KDM4B	chr19	5131088	5131188	+	1	0	100
Laptm5	chr1	30758842	30758942	-	1132	0	100
Laptm5	chr1	30746932	30747032	-	170	0	100
MBD2	chr18	54219731	54219831	-	7993	0	100
MMAA	chr4	145625973	145626073	+	1334	0	100
NFKBIE	chr6	44263240	44263340	-	4114	0	100
PAGR1	chr16	29816457	29816557	+	648	0	100
PGLYRP4	chr1	153343782	153343882	-	1888	0	100
PHPT1	chr9	136847953	136848053	+	294	0	100
PLLP	chr16	57260595	57260695	-	33538	0	100
PYROXD2	chr10	98391150	98391250	-	5340	0	100
SPIB	chr19	50419764	50419864	+	2396	0	100
STAC3	chr12	57249092	57249192	-	163	0	100
STAG3	chr7	100210960	100211060	+	880	0	100
UNC13C	chr15	54583777	54583877	+	3811	0	100
WDR91	chr7	135188773	135188873	-	142	0	100
GMNN	chr6	24779789	24779889	+	1780	1	100
KIFAP3	chr1	170071492	170071592	-	17114	15	100
BAG2	chr6	57172604	57172704	+	27958	48	100
GPR15	chr3	98536538	98536638	-	2731	6	100
TPRG1	chr3	189308204	189308304	`+	31247	100	100
MAP7D2	chrX	20056827	20056927	-	18778	103	99
NDUFAF4	chr6	96897383	96897483	-	6281	53	99
SERINC2	chr1	31423625	31423725	+	12442	105	99
SMG9	chr19	43748774	43748874	-	16446	212	99
SUPV3L1	chr10	69202538	69202638	+	1478	22	99
DHRS7	chr14	60210465	60210565	-	6814	189	97
MFSD14A	chr1	100077016	100077116	+	1161	39	97

NSMCE1	chr16	27243237	27243337	-	1676	59	97
JUP	chr17	41771828	41771928	-	640	27	96
DENND6B	chr22	50314503	50314603	-	1203	66	95
ICA1	chr7	8128778	8128878	-	7951	454	95
TMSB10	chr2	84905617	84905717	+	315135	19612	94
17ORF49	chr17	7015451	7015551	+	31898	2012	94
TIMD4	chr5	156922249	156922349	-	47152	3290	93
ACTB	chr7	5530538	5530638	-	15718	1321	92
2-Mar	chr1	220770439	220770539	+	1641	141	92
ADGRE1	chr19	6926293	6926393	+	22615	1981	92
ELMO1	chr7	36951258	36951358	-	112177	11189	91
UPB1	chr22	24503394	24503494	+	97503	10501	90
PTPN6	chr12	6954041	6954141	+	3993	698	85
DOCK2	chr5	169746401	169746501	+	20428	3811	84
TEKT4	chr2	94873323	94873423	+	2148	446	83
TYMP	chr22	50526939	50527039	-	97	21	82
CTSH	chr15	78939164	78939264	-	1373	428	76
MSC	chr8	71843775	71843875	-	2595	826	76
CD27	chr12	6450337	6450437	+	568	183	76
DR1	chr1	93346386	93346486	+	4733	1858	72
PLEK	chr2	68388968	68389068	+	77406	31720	71
NFKBIE	chr6	44260521	44260621	-	507	225	69
TNFRSF4	chr1	1213035	1213135	-	279	147	65
ANXA6	chr5	151124311	151124411	-	22068	13327	62
ANXA11	chr10	80201540	80201640	-	1496	1277	54
MAP2k2	chr19	4115016	4115116	-	16	15	52
CD19	chr16	28932342	28932442	+	9	43	17
MRPS17	chr7	55953127	55953227	+	96	1579	6
AICDA	chr12	8611097	8611197	-	0	0	0
ASAP1	chr8	130358572	130358672	-	0	0	0
ASCL1	chr12	102958440	102958540	+	0	0	0
BATF	chr14	75515759	75515859	+	0	0	0
BBC3	chr19	47228468	47228568	-	0	0	0
BCHE	chr3	165830479	165830579	-	0	150	0
CD70	chr19	6590688	6590788	-	0	0	0
CETP	chr16	56971286	56971386	+	0	0	0
CNFN	chr19	42387393	42387493	-	0	0	0
COG4	chr16	70483536	70483636	-	0	0	0
ECHS1	chr10	133373663	133373763	-	0	0	0
ENTPD2	chr9	137050661	137050761	-	0	0	0
FAM78A	chr9	131278122	131278222	-	0	0	0
ICA1	chr7	8176394	8176494	-	0	598	0
IRF2BP2	chr1	234610120	234610220	-	0	0	0
MICAL1	chr6	109454191	109454291	-	0	0	0
MRPS26	chr20	3046308	3046408	+	0	0	0
MYBL2	chr20	43710949	43711049	+	0	0	0
NUDT8	chr11	67629291	67629391	-	0	0	0

ORMDL1	chr2	189783966	189784066	-	0	0	0
PARP10	chr8	143978227	143978327	-	0	0	0
PIF1	chr15	64823953	64824053	-	0	0	0
RPL26	chr17	8384002	8384102	-	0	0	0
TFF2	chr21	42347644	42347744	-	0	0	0
TSPAN2	chr1	115073138	115073238	-	0	0	0
UNC13C	chr15	54408988	54409088	+	0	0	0
USP39	chr2	85628866	85628966	+	0	0	0

omprising 95 TSS
;. Weak support

CONFIRMED	Comments
YES	
YES	
YES	
YES	
YES	
YES	weak support
YES	
YES	
YES	
YES	
YES	
YES	1 of 3 TSS
YES	weak support
YES	1 of 2 TSS
YES	1 of 2 TSS
YES	
YES	
YES	1 of 2 TSS
YES	
YES	
YES	
YES	
YES	
YES	
YES	
YES	
YES	1 of 2 TSS
YES	
YES	
YES	
YES	
YES	
YES	
YES	
YES	
YES	
YES	
YES	
YES	
YES	

YES	
YES	
YES	
YES	1 of 3 TSS
YES	control
YES	
YES	
YES	control
YES	
YES	
YES	
YES	
YES	
YES	
YES	
YES	
YES	
YES	
YES	
YES	
YES	
YES	1 of 2 TSS
YES	
YES	
YES	
YES	weak support
NO	
NO	
NO	
NO	
NO	
NO	
NO	
NO	
NO	
NO	
NO	
NO	
NO	
NO	
NO	
NO	1 of 3 TSS
NO	
NO	
NO	
NO	
NO	

NO	
NO	
NO	
NO	
NO	
NO	
NO	1 of 2 TSS
NO	
