# Supplementary material for: Identification of high-confidence human poly(A) RNA isoform scaffolds using nanopore sequencing
Source: RNA. 2022 Feb;28(2):162–76. doi: 10.1261/rna.078703.121 (PMC8906549; doi:10.1261/rna.078703.121)
Supplement: Supplemental Material [file supp_078703.121_Supplemental_Table_S5.pdf]

Gene	TSS (chr:pos)	strand	primer	primer start
ACTB	chr7:5530588	-	ACGAGCGCGGCGATATCA	chr7:5,529,632
ADGRE1	chr19:6,926,343	+	AGCCCATAACCAAAGGCACAGA	chr19:6926538
AICDA	chr12:8,611,147	-	GTAAAGAAACTTCCTCCGGTTCATCAAGA	chr12:8606982
PHPT1	chr9:136,847,950	+	GTCCCCTGCCCCTTCGGCT	chr9:136848162
AMBRA1	chr11:46,483,148	-	TACTGGCCACAGCAGACTGTCCA	chr11:46443560
ANXA11	chr10:80,201,627	-	CCACCAGCTTATTTGGTATCTCAAAGACA	chr10:80201458
ANXA6	chr5:151,124,361	-	CATTGCTGCGGTGCGTGATGAT	chr5:151122964
ASAP1	chr8:130358622	-	CCGGCGGCGGCAGCT	chr8:130358425
ASCL1	chr12:102958490	+	GGTGGCAAAGCCCAGGTTGA	chr12:102958664
BAG2	chr6:57172654	+	TCTTGCTCAACAGCAGTTGCTGC	chr6:57182073
BATF	chr14:75515809	+	ACCCCTGCGTCCTCCTCA	chr14:75522535
BBC3	chr19:47228518	-	ACACTGCCGAGGGCACCA	chr19:47228326
BCHE	chr3:165830529	-	AGACAAATTCAATTTCATAGCCATGCATCACT	chr3:165829614
BCL2A1	chr15:80017784	-	TTAGGTTCAAACTTCTTTACAAAGCCA	chr15:79961143
BLK	chr8:11538235	+	CCATGGGACAACCAGGGCTGT	chr8:11538371
C17ORF49	chr17:7015501	+	TTCTGGGACCCCTTCCACGGAT	chr17:7015672
CCDC12	chr3:46927276	-	CTGCCAGGGCCCCTGAGCCT	chr3:46927083
CCR10	chr17:42682440	-	CCAGCGCAGCCACGGTC	chr17:42680479
CD19	chr16:28932392	+	AGATGGCCAGGGGCCTCATGT	chr16:28932498
CD27	chr12:6450387	+	ATCGGAGCTGCACAGGGATCTT	chr12:6450920
CD70	chr19:6590738	-	GTGTGATTCAGCTGCAGCTCAGCTA	chr19:6590105
CETP	chr16:56971336	+	CTGAGCATGAGGCGGCCAT	chr16:56973488
CNFN	chr19:42387443	-	CAGAGGGCGCAGGGCA	chr19:42387187
COG4	chr16:70483586	-	AAGCTGTTGATCCAAGGCTGCACCT	chr16:70482757
CORO1B	chr11:67438697	-	CTACGCATCCCCGTTCTCCA	chr11:67438376
CRELD2	chr22:49925143	+	CTGCTAGTGAGCACTCGTCCACA	chr22:49925440
CTSH	chr15:78939214	-	TGGTGGTACTCCTCCGTACTGTAGGTCTT	chr15:78937389
DENND6B	chr22:50314560	-	GCTCCCCGACTCGGAGGA	chr22:50314412
DHRS7	chr:60210516	-	CTGGTAAGCCAGCTCCTCACCAA	chr14:60156079
DOCK2	chr5:169746451	+	GGATTCAAAGAGCCGTCTCATGGA	chr5:169747456
DR1	chr1:93346436	+	GTTGCCAGACGAGGAAGCCAT	chr1:93346666

ECHS1	chr10:133373713	-	GACCAGCGCCCTCCA	chr10:133373558
ELMO1	chr7:36951308	-	CTACTGTTGCCACCTTGGTCCA	chr7:36951083
ENTPD2	chr9:137050711	-	CCGAGCAGCACTTGGGTGGAA	chr9:137050483
FAM78A	chr9:131278172	-	GCCGCTCGCCGGTGA	chr9:131276761
FBXL15	chr10:102421141	+	TTCAGGACGTGTGGGAGCAGCAC	chr10:102421401
FHL2	chr2:105373980	-	AAACAAGGAAAGAAGTTGGGCCGA	chr2:105373822
FLOT2	chr17:28897042	-	CTCTGAGTGTCGGAGATACACCACCA	chr17:28888945
GEMIN7	chr19:45090911	+	AGCCACGGCTGAAGCCATCA	chr19:45090187
GMNN	chr6:24799839	+	AGTTCTGCTTCAAGCCCATTCA	chr6:24779920
GPR15 (antisense)	chr3:98536588	-	CCAGGGAGCTCACGCTGATTG	chr3:98532545
ICA1	chr7:8129102	-	AGCAGCAGGTCATCTTTGTCAGCA	chr7:8128079
IRF2BP2	chr1:234610170	-	CGAGCGGCGGGGCAT	chr1:234609974
JUP	chr17:41771878	-	CTTGCTGCTGACGGAGGGCA	chr17:41771735
KDM4B	chr19:5131138	+	GCCTCTAGCTCCTCACTGGGCA	chr19:5131319
KDM4B	chr19:5131138	+	GCACCACGTACAGCATGGGGA	chr19:5131357
KIFAP3	chr1:170071542	-	CCATTTCTCCAAGAATGGTAGCTTCCA	chr1:170,055,345
Laptm5	chr1:30758892	-	CAAAAAGTTGGAATTAATAAGCCACAACTCT	chr1:30,758,720
Laptm5a	chr1:30746982	-	GCCACCTCTACTGAGTGCTCGA	chr1:30,742,506
MAP2k2	chr19:4115066	-	GCTGATCTCCCCGTCACTGTAGAA	chr19:4,110,527
MAP7D2	chrX:20056877	-	CTGGGGAATAGGATATTGCCACGGT	chrX:20,052,883
MARC2	chr1:220770489	+	CTACACTACCAATTAGGAGTTCATCCCA	chr1:220,780,051
MBD2	chr18:54219781	-	AGGCTTGCTTCTGAACTTCTTACCACTT	chr18:54,205,127
MFSD14A	chr1:100077066	+	GCTACTGCCCCAGCAGCCCACA	chr1:100,078,483
MICAL1	chr6:109454241	-	GAGCTGGTCCTTGATCTTGTGGTACT	chr6:109454031
MMAA	chr4:145626023	+	CTGCTCATGTAACTCAGCATCTTTTCCT	chr4:145,626,208
MRPS17	chr7:55953177	+	GTGAGCAAAGTAGGTTTTCCGCTT	chr7:55,954,941
MRPS26	chr20:3046358	+	CTCCTGCCGCAGCCTCGCTAT	chr20:3046635
MSC	chr8:71844038	-	CACTGGGTGCACGTAGCCGTT	chr8:71843651
MYBL2	chr20:43710999	+	GGCCTGCCATGGCTGGGA	chr20:43,711,291
NDUFAF4	chr6:96897433	-	GTCAGGCTCTGCGTCCATCGT	chr6:96897307
NFKBIE	chr6:44260571	-	CCCCGCTCTTGGGTTTCCACA	chr6:44260227
NFKBIEa	chr6:44263290	-	GGAGCAAGGAGACAGTGCAGCAT	chr6:44263157

NSMCE1	chr16:27243287	-	AGCAACCAATGCAGAGGCGA	chr16:27241815
NUDT8	chr11:67629341	-	CCTGAGCAATGGGGATAAGGAAGAGA	chr11:67629220
ORMDL1	chr2:189784016	-	TTCTTTCTGAATACTCAATGTGGAAGGTA	chr2:189783091
PAGR1	chr16:29816867	+	AGAGCCTGGAGGCCGGA	chr16:29816617
PARP10	chr8:143978277	-	CGACACGCGCTCCACCT	chr8:143978067
PGLYRP4	chr1:153343832	-	TGCCTGTGTGATCCACGCAGA	chr1:153343576
PIF1	chr15:64824003	-	GTAGTGTCCGGAACCCGGGT	chr15:64823826
PLEK	chr2:68389018	+	ATCATATGCCTTGGCACCTGGT	chr2:68389152
PLLP	chr16:57260645	-	TGATGTAGAGAACGGTGGCGCT	chr16:57258545
PTPN6	chr12:6954091	+	GGAGATGGACACACAGACTCAAGT	chr12:6954268
PYROXD2	chr10:98391200	-	GACCGGGTGTCCAGCTGAGAGA	chr10:98391030
RPL26	chr17:8384052	-	TCCAAGGCCGCGTTTCCAA	chr17:8383853
SERINC2	chr1:31423675	+	AGTCGATGTGGCCCTGCAGGA	chr1:31424743
SMG9	chr19:43748824	-	GCTGAAGAGGGGAGAAATCTGAGTCT	chr19:43748689
SPIB	chr19:50419814	+	ACAGCTGAAGTGTGGCCCGTCGA	chr19:50419970
STAC3	chr12:57249142	-	ACATAGGACTGACAGTGTTCATGGATGT	chr12:57248734
STAG3	chr7:100211010	+	GTGGACCCAAGAACCTTGACCTCT	chr7:100211481
SUPV3L1	chr10:69202588	+	GCAGACAAAATACTGCCCATCA	chr10:69202914
TEKT4	chr2:94873373	+	CATCTCGCAGGTCTCCTTGTGCT	chr2:94873998
TFF2	chr21:42347694	-	GCACTTCAAAGATGAAGTTGGAGAAGCA	chr21:42347515
TIMD4	chr5:156922299	-	GCATTTCATTCTTCATTGACATGGGTA	chr5:156922180
TMSB10	chr2:84905292	+	GCGATTTCCCCCATGTCTGGT	chr2:84906049
TNFRSF4	chr1:1213085	-	TTGCAGGCCTGGTTGTCGCCT	chr1:1212650
TPGR1 (TPRG1)	chr3:189308254	+	GCATAAGGAACTTCAGTGGACCAT	chr3:189310481
TSPAN2	chr1:115073188	-	GCCGATCCAGCCAGCT	chr1:115072984
TYMP	chr22:50526989	-	GGGCTTGTCCATGGCGGTCA	chr22:50526676
UNC13C	chr15:54583827	+	ACGGCATCTTTGGAGGAACGA	chr15:54581839
UNC13C	chr15:54409038	+	TTTCCCATGTTCAGCTCTTGAGGAA	chr15:54415008
UPB1	chr22:24503444	+	TCTCTCGTACAGAAGGCAAAGGGCA	chr22:24510776
USP39	chr2:85628916	+	TGGACCAACAAGAACATGATATCCCCT	chr2:85630821
WDR91	chr7:135188823	-	TGTTCTCATCATAGCTGAACTCCACAGA	chr7:135188462
ICA1	chr7:8176444	-	AGATTGCATCTGCTCGCTCCAAGA	chr7:8158555
