# Supplementary material for: Identification of high-confidence human poly(A) RNA isoform scaffolds using nanopore sequencing
Source: RNA. 2022 Feb;28(2):162–76. doi: 10.1261/rna.078703.121 (PMC8906549; doi:10.1261/rna.078703.121)
Supplement: Supplemental Material [file supp_078703.121_Supplemental_Table_S6.pdf]

Comparison of TSS predictions from various RNA-Seq methods with orthogonal experimental GM12878 TSS evidence from ENCODE on GRCh38 primary assembly. For each of the RNA-Seq methods, the 5' most base was extended by 25 bases on either end and merged with overlapping TSS regions on the same strand to define TSS predictions. If the TSS prediction was within 25 bases of the ENCODE CAGE mappings (ENCFF103DEY, ENCFF260HMC) on the same strand, or overlapped Pol-II narrow peaks (ENCFF303DQB, ENCFF324NBP), or DNase-seq broad peaks (ENCFF235KUD, ENCFF491BOT), the predictions are considered confirmed. The strand read column is the number of molecules sequenced. The isoform predictions column is the number of StringTie2 or FLAIR models use in computing the TSS predictions, with both the number and rate of confirmed merged TSS prediction given for each evidence independently and combined.

RNA set	Strand reads	Isoform predictions	CAGE confirmed TSS		DNase-1 confirmed TSS		pol2 confirmed TSS		combined confirmed TSS	
untreated	3,685,619	-	19,470	(5.3%)	8,151	(2.2%)	138,439	(37.5%)	145,855	(39.5%)
untreated - FLAIR	3,685,619	35,968	10,765	(44.9%)	1,006	(4.2%)	17,151	(71.5%)	17,934	(74.7%)
untreated - StringTie2	3,685,619	46,979	6,430	(28.1%)	900	(3.9%)	14,062	(61.4%)	14,943	(65.3%)
treated	3,878,369	-	18,166	(4.5%)	8,563	(2.1%)	142,909	(35.5%)	150,583	(37.4%)
cap-adapted	545,558	-	12,527	(37.5%)	1,425	(4.3%)	23,232	(69.6%)	24,726	(74.1%)
high-confidence scaffolds	170,095	-	8,071	(60.3%)	781	(5.8%)	11,818	(88.3%)	12,341	(92.2%)
cdna	19,152,167	-	19,171	(1.9%)	19,766	(2.0%)	225,140	(22.3%)	242,160	(24.0%)
cDNA - FLAIR	19,152,167	569,714	10,342	(7.2%)	3,335	(2.3%)	38,487	(27.0%)	41,266	(28.9%)
cDNA - StringTie2	19,152,167	157,918	5,020	(5.4%)	1,995	(2.1%)	21,730	(23.3%)	23,392	(25.1%)
