# Supplementary material for: Identification of high-confidence human poly(A) RNA isoform scaffolds using nanopore sequencing
Source: RNA. 2022 Feb;28(2):162–76. doi: 10.1261/rna.078703.121 (PMC8906549; doi:10.1261/rna.078703.121)
Supplement: Supplemental Material [file supp_078703.121_Supplemental_Table_S7.pdf]

Table of the human genome coverage by different sources of TSS markers for GM12878 cells

evidence	count	bases	genome
CAGE	24,135	3,623,130	0.1%
DNaseI-sec	84,811	29,636,264	1.0%
pol2	157,033	111,230,851	3.6%
combined	254,018	142,694,859	4.6%
